# The Sole DEAD-Box RNA Helicase of the Gastric Pathogen *Helicobacter pylori* Is Essential for Colonization

**DOI:** 10.1128/mBio.02071-17

**Published:** 2018-03-27

**Authors:** Lamya El Mortaji, Sylvie Aubert, Eloïse Galtier, Christine Schmitt, Karine Anger, Yulia Redko, Yves Quentin, Hilde De Reuse

**Affiliations:** a Institut Pasteur, Département de Microbiologie, Unité Pathogenèse de Helicobacter, ERL CNRS 6002, Paris, France; b Université Paris Diderot, Sorbonne Paris Cité, Cellule Pasteur, Paris, France; c Institut Pasteur, Imagopole, Plate-Forme de Microscopie Ultrastructurale, Paris, France; d Laboratoire de Microbiologie and Génétique Moléculaires, UMR5100, Centre de Biologie Intégrative, Centre National de la Recherche Scientifique, Université de Toulouse, Toulouse, France; New York University

**Keywords:** DEAD-box helicase, *Helicobacter pylori*, RNA degradation, flagellar motility, mouse model

## Abstract

Present in every kingdom of life, generally in multiple copies, DEAD-box RNA helicases are specialized enzymes that unwind RNA secondary structures. They play major roles in mRNA decay, ribosome biogenesis, and adaptation to cold temperatures. Most bacteria have multiple DEAD-box helicases that present both specialized and partially redundant functions. By using phylogenomics, we revealed that the *Helicobacter* genus, including the major gastric pathogen *H. pylori*, is among the exceptions, as it encodes a sole DEAD-box RNA helicase. In *H. pylori*, this helicase, designated RhpA, forms a minimal RNA degradosome together with the essential RNase, RNase J, a major player in the control of RNA decay. Here, we used *H. pylori* as a model organism with a sole DEAD-box helicase and investigated the role of this helicase in *H. pylori* physiology, ribosome assembly, and during *in vivo* colonization. Our data showed that RhpA is dispensable for growth at 37°C but crucial at 33°C, suggesting an essential role of the helicase in cold adaptation. Moreover, we found that a Δ*rhpA* mutant was impaired in motility and deficient in colonization of the mouse model. RhpA is involved in the maturation of 16S rRNA at 37°C and is associated with translating ribosomes. At 33°C, RhpA is, in addition, recruited to individual ribosomal subunits. Finally, via its role in the RNA degradosome, RhpA directs the regulation of the expression of its partner, RNase J. RhpA is thus a multifunctional enzyme that, in *H. pylori*, plays a central role in gene regulation and in the control of virulence.

## IMPORTANCE

We present the results of our study on the role of RhpA, the sole DEAD-box RNA helicase encoded by the major gastric pathogen *Helicobacter pylori*. We observed that all the *Helicobacter* species possess such a sole helicase, in contrast to most free-living bacteria. RhpA is not essential for growth of *H. pylori* under normal conditions. However, deletion of *rhpA* leads to a motility defect and to total inhibition of the ability of *H. pylori* to colonize a mouse model. We also demonstrated that this helicase encompasses most of the functions of its specialized orthologs described so far. We found that RhpA is a key element of the bacterial adaptation to colder temperatures and plays a minor role in ribosome biogenesis. Finally, RhpA regulates transcription of the *rnj* gene encoding RNase J, its essential partner in the minimal *H. pylori* RNA degradosome, and thus plays a crucial role in the control of RNA decay.

## INTRODUCTION

Post-transcriptional control is a major level of regulation of gene expression in every kingdom of life. Protein complexes designated RNA degradosomes are molecular machines specialized in the control of RNA decay; they have been identified in several bacterial species, although with various compositions (1). All the characterized RNA degradosomes comprise at least one DEAD-box RNA helicase, underlining the essentiality of the function of this enzyme in RNA unwinding to facilitate the access of the degradation enzymes to their target RNAs in this protein complex. The *Escherichia coli* RNA degradosome is composed of an essential endoribonuclease, RNase E, that is associated with the metabolic enzyme enolase, the exoribonuclease PNPase, and the RhlB DEAD-box RNA helicase (1). About half of the sequenced bacterial species lack an orthologue of RNase E and have instead RNase J, an enzyme that combines both endo- and exo-ribonuclease activities (2, 3).

The Gram-negative bacterium *Helicobacter pylori* expresses a unique RNase J protein that is essential for *in vitro* growth (4). *H. pylori* is a pathogen of major importance that colonizes the stomach of about half of the human population worldwide. Infection by *H. pylori* causes the development of gastroduodenal ulcers, mucosa-associated lymphoid tissue lymphoma, and gastric carcinoma (5, 6). Gastric cancer is responsible for about 800,000 deaths in the world every year. Virulence of *H. pylori* relies on its capacity to adapt to and persistently colonize the hostile acid stomach. *H. pylori* possesses a small genome (1.6 Mb) and a reduced number of transcriptional regulators, suggesting a major role for post-transcriptional regulation in this organism (7). We have demonstrated that *H. pylori* possesses a functional two-partner RNA degradosome composed of RNase J and RhpA, a DEAD-box RNA helicase (4). In addition, we observed that both proteins are associated with translating ribosomes and not with individual 30S to 50S ribosomal subunits (4). From a recent transcriptome sequencing analysis, we found that about 80% of the *H. pylori* mRNAs are at least twice as abundant in an RNase J-depleted strain (8). This indicates a major role of RNase J in the control of mRNA stability in *H. pylori*. In the present study, we investigated the function of the second partner of the *H. pylori* degradosome, the RhpA DEAD-box RNA helicase.

DEAD-box RNA helicases are ubiquitous enzymes that constitute the largest family of RNA helicases (9, 10). This family of RNA helicases is designated “DEAD-boxes” because their members contain a conserved motif with the amino acid sequence D-E-A-D (Asp-Glu-Ala-Asp). They use ATP to unwind short duplex RNAs and to remodel ribonucleoprotein complexes. In bacteria, these proteins are key to RNA metabolism and central in many essential cellular processes, with major roles in (i) RNA turnover, (ii) ribosome biogenesis, and (iii) translation initiation (9). Bacteria generally possess multiple DEAD-box RNA helicases that have versatile and partially overlapping functions. The two model bacteria *E. coli* and *Bacillus subtilis* express 5 and 4 DEAD-box helicases, respectively. In many bacteria, DEAD-box helicases are involved in adaptation to changing environments. The most frequently reported role is adaptation to cold shock with, at low temperature, either the induction of the production of a helicase and/or reduced growth of the mutants defective in one or more helicases (11). In *Listeria monocytogenes*, the CshA DEAD-box helicase is required for full motility at low temperatures (12, 13). In every organism, one or more helicases are involved in ribosome biogenesis or rRNA maturation. However, their role in ribosome biogenesis is not vital for growth at normal temperature, since *E. coli*, *B. subtilis*, and *L. monocytogenes* mutants deleted for every DEAD-box helicase are viable (13–16). DEAD-box helicases have also been found to regulate the expression of virulence traits in *L. monocytogenes* (hemolytic activity) (17) and in *Staphylococcus aureus* (quorum-sensing control) (18). Finally, as a core component of RNA degradosomes, DEAD-box helicases have a global role in the control of RNA processing and mRNA turnover in several bacterial species, including *E. coli*, *S. aureus*, *B. subtilis*, and *H. pylori* (1, 4, 19, 20).

The multiplicity of DEAD-box helicases in the bacterial organisms mentioned above is believed to correspond, at least in part, to functional specialization. In contrast, *H. pylori* possesses only one DEAD-box RNA helicase, RhpA. Therefore, this organism appears to be an excellent study model to explore, for the first time, the function of a sole DEAD-box helicase and to ask questions regarding its essentiality and functional specialization. In the present study, we showed that the sole DEAD-box helicase of *H. pylori* is not essential for growth under normal conditions but is vital at lower temperatures. Moreover, RhpA plays a role in the maturation of rRNA and thus ribosome biogenesis. Finally, RhpA is required for full motility and for colonization of the mouse model by *H. pylori*. Thus, in *H. pylori* RhpA fulfills the functions of the multiple DEAD-box helicases and importantly regulates essential virulence determinants.

## RESULTS

### Phylogeny of DEAD-box helicases in *Epsilonproteobacteria*.

**FIG 1:**
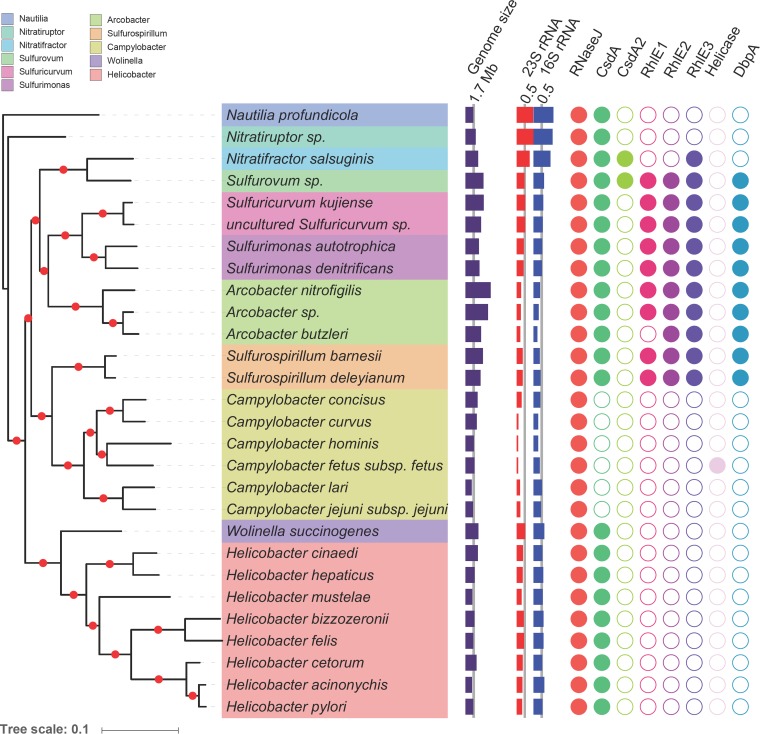
Distribution of RNase J and DEAD-box helicases among *Epsilonproteobacteria*. (Left) The phylogenetic tree of *Epsilonproteobacteria* species (constructed as described in Materials and Methods). The tree was rooted according to the methods described in reference 57. The red dots indicate branches with 100% bootstrap support. The tree leaves are colored according to the taxonomy. (Right) The purple histogram indicates genome sizes, with a 1.7-Mb scale line added; the red and blue histograms report the GC frequencies of 23S and 16S rRNAs, with 0.5 scale lines added, and the filled and empty circles correspond to the presence or absence of proteins, respectively. Colors refer to protein families. The RNase J proteins and DEAD-box helicases (CsdA, CsdA2, RhlE1, RhlE2, RhlE3, helicase, and DbpA) are also listed in Tables S5 and S6. The helicase families correspond to those identified in Fig. S2, according to the placement of the *E. coli* helicase proteins in the tree. *E. coli* DEAD protein has been reassigned CsdA, for **c**old **s**hock **D**EAD-box protein **A**. The CsdA family comprises two paralogues, one of which is homologous to the RhpA protein of *H. pylori* and the other is CsdA2. The RhlE DEAD-box helicase family decomposes into three subfamilies; “helicase” refers to an unclassified DEAD-box protein.

We previously discovered the existence of a minimal RNA degradosome in *H. pylori* composed of the essential RNase J ribonuclease and of a newly identified DEAD-box RNA helicase that we designated RhpA (4). *H. pylori* is one of the most prominent species of the *Epsilonproteobacteria* class, which contains other important pathogens such as *Campylobacter jejuni*. Interestingly, orthologues of RNase J are found in all *Epsilonproteobacteria* species, while no RNase E/G orthologues are detected (Fig. 1). The tree obtained with RNase J (see Fig. S1 in the supplemental material) suggests that the *rnj* genes were vertically transmitted from the last common ancestor of *Epsilonproteobacteria* species. We thus decided to investigate for the first time the distribution and phylogeny of the DEAD-box helicases among the *Epsilonproteobacteria*. In contrast to the other organisms analyzed to date, the data we obtained revealed the presence of a sole DEAD-box helicase that is conserved in every sequenced *H. pylori* strain as well as in each non-*pylori Helicobacter* species (Fig. 1). More surprisingly, we observed that all of the *Campylobacter* species lack a DEAD-box helicase with the exception of *C. fetus*, which contains one such helicase that is phylogenetically distant from that of *Helicobacter* species and presents characteristics of horizontal gene transfer (Fig. S2). Among the *Epsilonproteobacteria*, we found a correlation between the absence of helicase and a lower GC content of 23S and 16S rRNAs (Fig. 1). As expected, more-distant free-living *Epsilonproteobacteria* with larger genomes, like *Sulfurospirillum*, possess multiple helicases, except for the thermophilic species *Nitratiruptor* sp. and *Nautilia profundicola* (Fig. 1). Finally, with the exception of the majority of the *Campylobacter* species, every *Epsilonproteobacteria* species, including the *Helicobacter* species, possesses a similar helicase that is distantly related to the CsdA cold shock helicase family, suggesting the presence of such a helicase in their common ancestor (Fig. S2). Thus, *H. pylori* is an excellent model organism to study the role of a sole DEAD-box helicase.

10.1128/mBio.02071-17.1 FIG S1  Phylogenic tree of RNase J from *Epsilonproteobacteria*. (Left) The phylogenetic tree of RNase J homologues, constructed as described in Materials and Methods. Red dot sizes are proportional to the extent of bootstrap support. The proteins at tree leaves are replaced by species names and are colored according to the taxonomy (see Table S6). If the less-supported nodes were excluded, the RNase J tree was congruent with the species tree, suggesting that the *rnj* genes were vertically transmitted from the last common ancestor of *Epsilonproteobacteria*. (Right) Diagram of the primary structures of RNase J homologues, showing the conserved Pfam domains. Download FIG S1, DOCX file, 0.3 MB. Copyright © 2018 El Mortaji et al. 2018 El Mortaji et al. This content is distributed under the terms of the Creative Commons Attribution 4.0 International license.

10.1128/mBio.02071-17.2 FIG S2  Phylogenic tree of the DEAD-box helicases in *Epsilonproteobacteria*. (Left) The phylogenetic tree of DEAD-box helicase homologues, constructed as described in Materials and Methods. The tree was rooted between the related proteins DbpA and CsdA/RhlE as described in reference 58. Red dot sizes are proportional to the extent of bootstrap support. The proteins at tree leaves are replaced by species names and are colored according to the helicase families (see Table S5). The *E. coli* DEAD-box helicase tree leaves are colored orange with the family name. The *E. coli* proteins SrmB and RhlB do not have orthologues in *Epsilonproteobacteria*. The topology of the CsdA subtree is very similar to that of the species tree, suggesting that a gene encoding the CsdA protein homologue was present in the last common ancestor of *Epsilonproteobacteria* and that it was vertically transmitted to its descendants. The species *Nitratifractor salsuginis* and *Sulfurovum* sp. encode DEAD-box helicase paralogues. Their distant location on the tree suggests that an HGT was responsible for the acquisition of a new copy of this gene in the last common ancestor of *Nitratifractor salsuginis* and *Sulfurovum* sp. (Right) Diagram of primary structures of DEAD-box helicase homologues, showing the conserved Pfam domains. Download FIG S2, DOCX file, 1.8 MB. Copyright © 2018 El Mortaji et al. 2018 El Mortaji et al. This content is distributed under the terms of the Creative Commons Attribution 4.0 International license.

### RhpA, the sole DEAD-box helicase of *H. pylori*, is dispensable for growth.

**FIG 2:**
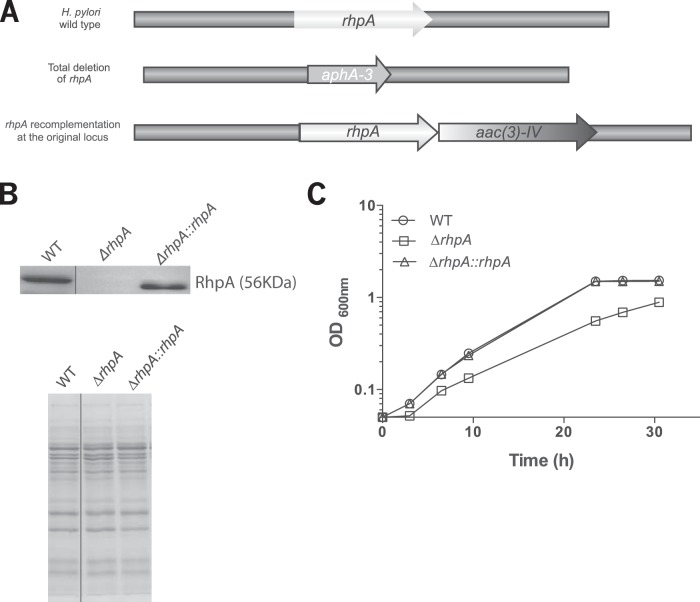
Construction, validation, and growth analysis of an *H. pylori rhpA* deletion mutant and a complemented strain. (A) A total *rhpA* deletion was created in *H. pylori* strain X47-2AL by insertion of the *aphA-3* gene that encodes aminoglycoside 3′-phosphotransferase, which confers kanamycin resistance. From this mutant, a complemented strain was obtained by reintroducing, at the original locus in *cis*, a wild-type *rhpA* copy and selecting for gentamicin resistance conferred by the *aac(3)-IV* gene. (B) Western blotting of whole-cell extracts of X47-2AL wild-type (WT), *ΔrhpA* mutant, and *rhpA* recomplemented strains with anti-RhpA polyclonal antibodies. A Coomassie-stained SDS-PAGE gel is presented below (a loading control). Spliced sites of the original gel are marked by a thin line. (C) Growth of X47-2AL wild-type, *ΔrhpA* deletion mutant, and the *rhpA*-complemented strains in BHI medium.

To investigate the role of RhpA in *H. pylori* physiology, we decided to construct an *H. pylori* mutant strain carrying a complete deletion of *rhpA* in the X47-2AL background (21, 22). This mutant was perfectly viable, indicating that *rhpA* is not an essential gene in *H. pylori*. A complemented strain was also obtained by reintroducing a wild-type *rhpA* copy at the original locus in *cis* (Fig. 2A). These strains were grown in brain heart infusion (BHI) medium at 37°C to determine their growth rate and to monitor RhpA expression levels. Growth of the *ΔrhpA* mutant was found to be moderately affected under our experimental conditions, with a doubling time of 376 min (±25 min [standard error of the mean doubling time]) compared to the wild-type and complemented strains, which had doubling times of 242 min (±12 min) and 243 min (±23 min), respectively (Fig. 2C). Comparable growth reduction was observed in *ΔrphA* mutants constructed in two other *H. pylori* backgrounds (strains 26695 [23] and B128 [24]). Western blot analysis of total extracts with an anti-RhpA polyclonal antibody (4) confirmed the absence of RhpA production in the *ΔrhpA* mutant strain and detected similar amounts of RhpA protein in the wild-type and complemented strains (Fig. 2B). Finally, examination of cell morphology by scanning electron microscopy indicated that the X47-2AL *ΔrhpA* mutant and complemented strains were indistinguishable from the wild type.

### The RhpA helicase is required for colonization by *H. pylori* in the mouse model*.*

**FIG 3:**
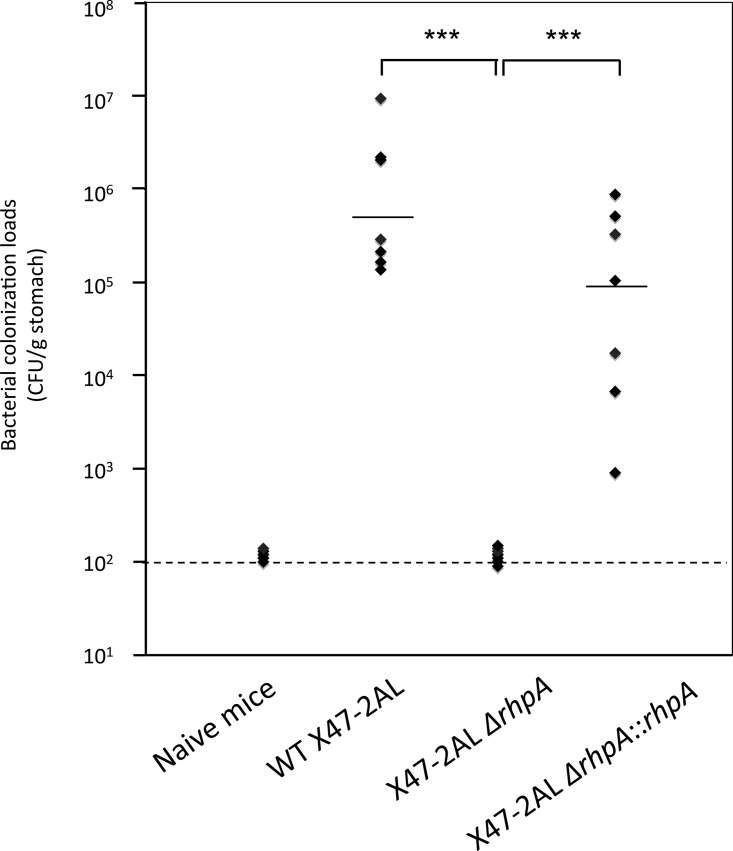
The X47-2AL Δ*rhpA* mutant is defective in mouse stomach colonization. Each point corresponds to the colonization load for one mouse, 1 month after infection with the strain indicated. Naive mice were inoculated with culture medium as a negative control. Horizontal bars represent the geometric means of the colonization load for the wild-type (WT), the *ΔrhpA* mutant, and the *rhpA*-complemented strain. The results presented correspond to those of one representative experiment of two conducted. The detection limit is shown by a dashed horizontal line and corresponds to 100 CFU per g of stomach. ***, the geometric mean was significantly different (*P* ≤ 0.001).

In order to determine whether the sole DEAD-box helicase of *H. pylori* is required for *in vivo* colonization, we tested the ability of the *ΔrhpA* mutant to colonize mouse stomach. To this end, seven NMRI mice were orogastrically infected with the *H. pylori* X47-2AL wild-type strain, with the isogenic *ΔrhpA* deletion mutant, or with the *rhpA* complemented strain (Fig. 3). Four weeks after infection, the colonization loads of the mice were assessed by quantitative cultures of stomach homogenates. Bacterial enumeration showed that the *ΔrhpA* mutant was completely deficient in its ability to colonize mice, while the geometric mean of colonization by the parental wild-type strain reached 4 × 10^5^ CFU/g stomach. The colonization ability of the complemented strain was restored to a level close to that of the wild-type strain.

Previous work by Bijlsma et al. suggested that an insertion mutant for the gene encoding the DEAD-box RNA helicase of *H. pylori* strain 1061 presents a urease-negative phenotype (25). Since urease activity is essential for stomach colonization by *H. pylori* (26), we investigated in the X47-2AL strain background whether the *ΔrhpA* mutant presented a similar defect in the bacterial urease pathway. Western blot analysis of total extracts showed that the amounts of both urease structural subunits UreA and UreB were not affected in the helicase mutant compared to amounts in the wild-type strain, indicating no defect in the expression of the two structural subunits (Fig. S3A). As an additional confirmation, urease activity determinations revealed that the X47-2AL *rhpA* deletion mutant retained wild-type urease activity levels similar to the wild type (Fig. S3B), and the same observation was made with a *ΔrhpA* mutant in another background, B128.

10.1128/mBio.02071-17.3 FIG S3  Synthesis of the urease structural subunits and urease activity are not controlled by RhpA. (A) Western blotting with anti-UreA and anti-UreB polyclonal antibodies of total extracts from X47-2AL wild-type strain, the *ΔrhpA* deletion mutant, or the *rhpA* complemented strain. Similar amounts of the two urease structural subunits, UreA and UreB, were synthesized by these three strains. (B) Urease activity of strain X47-2AL and the isogenic *ΔrhpA* deletion mutant. The urease activity was determined for whole cells at pH 5.0 and pH 7.0 by measuring the ammonia production after addition of 5 mM urea. Each strain produced similar amounts of ammonia, indicating similar urease activities. The data correspond to the mean values of three independent experiments, and error bars represent the standard deviations. Download FIG S3, DOCX file, 0.1 MB. Copyright © 2018 El Mortaji et al. 2018 El Mortaji et al. This content is distributed under the terms of the Creative Commons Attribution 4.0 International license.

Taken together, these data showed that *rhpA* is essential for gastric colonization in a mouse model and that the colonization defect observed is not due to a reduction in urease activity.

### The helicase mutant is affected in motility.

**FIG 4:**
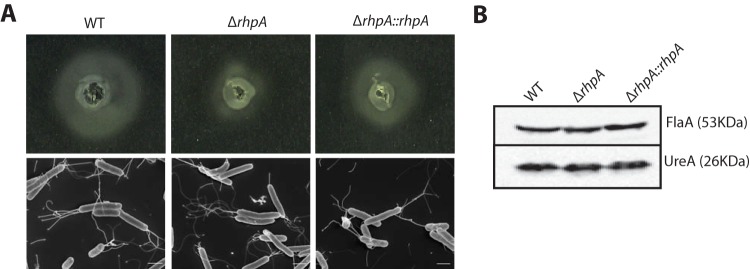
An *H. pylori* mutant deficient in RhpA is deficient in motility despite intact flagella and wild-type (WT) amounts of the major flagellin, FlaA. (A, upper panel) Motility of the X47-2AL *ΔrhpA* deletion mutant was diminished on soft agar plates compared to the X47-2AL wild-type strain and an isogenic *rhpA*-complemented strain. (Lower panel) Based on scanning electron microscopy, no visible differences in the aspect or number of flagella were observed with the three strains. Scale bar, 1 μm. (B) Levels of the major flagellin protein FlaA were determined by Western blotting using anti-FlaA specific antibodies. Equal amounts of total extract from X47-2AL wild-type strain, the isogenic *ΔrhpA* deletion mutant, or the isogenic *rhpA*-complemented strain were analyzed. UreA served as a loading control. Normalization indicated there were no differenced in FlaA production in the tested strains.

Numerous factors can influence the establishment of *H. pylori* colonization either directly or indirectly (27). One factor is the ability of *H. pylori* to be fully motile. Thus, the motility of the X47-2AL *ΔrhpA* mutant strain was examined by stab inoculation on soft agar plates supplemented with 10% fetal calf serum and incubation at 37°C for 4 days (Fig. 4A, upper panel). Interestingly, the *ΔrhpA* mutant strain showed a reproducible motility defect, with a motility halo diameter that was about half that of the wild-type strain. The motility level was restored in the complemented strain and almost reached that of the parental strain. To further determine whether this decreased motility was due to defective flagella, the morphology of the different strains was examined by scanning electron microscopy (Fig. 4A, lower panel). Similar to the wild-type strain, both *rhpA* deletion and complemented mutant strains presented intact unipolar flagella in comparable numbers. The level of the major flagellar protein FlaA was further determined in these strains by Western blotting with anti-FlaA polyclonal antibodies (Fig. 4B). Quantification of the immunoreactive bands revealed no significant difference between the wild-type, Δ*rhpA* mutant, and complemented strains. Together, these data showed that the RhpA-deficient strain was affected in motility despite normal flagella and FlaA production.

### Cold temperature sensitivity of the helicase mutant.

**FIG 5:**
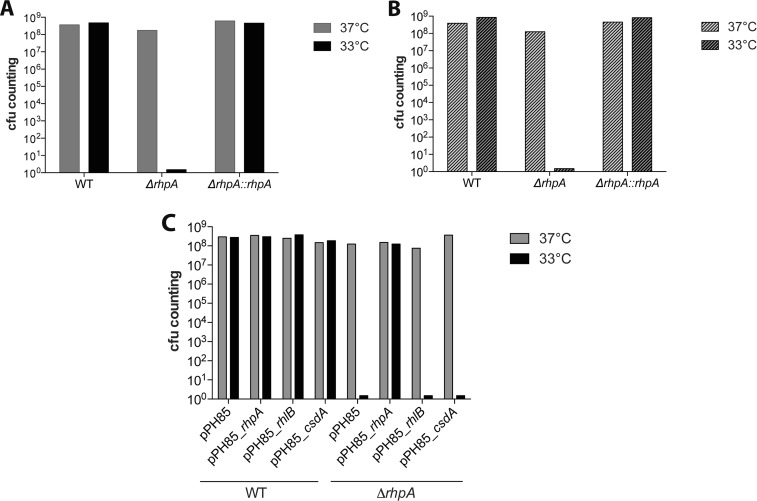
RhpA is essential for growth of *H. pylori* at 33°C and is not functionally complemented by its *E. coli* homologues, RhlB or CsdA. Amounts of viable bacteria of *H. pylori* wild-type (WT), *ΔrhpA* mutant, and complemented strains were measured after incubation in BHI medium during 16 h of growth at 37°C or at 33°C. (A) Wild-type strain X47-2AL, the *ΔrhpA* mutant, and the complemented mutant were incubated and colonies were enumerated on plates incubated at the same temperature (either 33°C or 37°C, as indicated). (B) Wild-type strain X47-2AL, the *ΔrhpA* mutant, and the complemented mutant were first incubated at 33°C and subsequently spotted on plates at the temperature indicated in the legend, i.e., at either 33°C or 37°C. The lower temperature of 33°C completely inhibited growth of the *ΔrhpA* mutant, but this growth could be rescued by subsequent incubation at 37°C. (C) Viable bacteria of *H. pylori* B128 wild type and of the *ΔrhpA* mutant either carrying an empty vector (pPH85) or a vector expressing *csdA*, *rhlB*, or *rhpA* under control of an IPTG-inducible vector were enumerated. Strains were plated on blood agar medium and incubated at either 33°C or 37°C for 5 days. The lower temperature inhibited Δ*rhpA* mutant growth on plates, and growth was restored when *rhpA* was expressed from a plasmid (pPH85_*rhpA*). Neither CsdA nor RhlB was able to rescue the cold-sensitive phenotype of the Δ*rhpA* mutant strain at 33°C (see also Fig. S4).

DEAD-box helicases have been shown to be important for several cellular functions, including adaptation to cold temperature. To test whether the *H. pylori* sole DEAD-box helicase is involved in this process, we first established that 33°C is the lowest temperature at which *H. pylori* growth is identical to that at 37°C. Then, growth of the wild-type X47-2AL strain, isogenic helicase mutant, and complemented strain was monitored at 33°C. As shown in Fig. 5A, multiplication of the *ΔrhpA* mutant strain was completely abolished at 33°C. To investigate whether 33°C is a lethal temperature for the *ΔrhpA* mutant strain or if the observed growth inhibition was reversible, we incubated the three strains at 33°C and subsequently spotted them on plates incubated at either 33°C or 37°C (Fig. 5B). Prior incubation at 33°C followed by incubation at 37°C did not alter the viability of the Δ*rhpA* mutant, indicating that this mutant is able to recover from “cold” (or lower-temperature) exposure. In parallel, the three strains presented no difference in growth at a higher temperature (39°C).

Thus, the RhpA helicase is required for the adaptation of *H. pylori* to growth at the lower temperature of 33°C.

### Do the *E. coli* RhlB and CsdA DEAD-box RNA helicases phenotypically complement the *H. pylori rhpA* mutant?

RhpA is a core component of the *H. pylori* RNA degradosome, and according to the phylogeny of *Epsilonproteobacteria* presented above, RhpA is related to the CsdA family of DEAD-box helicases. In the Gram-negative model organism *E. coli*, CsdA plays a major role during cold shock adaptation, while another helicase, RhlB, is involved in RNA decay (9, 10). We therefore reasoned that one or both of these helicases might complement the growth phenotype of the *H. pylori ΔrhpA* mutant. To test this hypothesis, three derivatives of the pILL2157-derived pPH85 *E. coli-H. pylori* shuttle vector were constructed and, under control of an isopropyl-β-d-thiogalactopyranoside (IPTG)-inducible promoter, expressed either *rhlB*, *csdA*, or *rhpA* as a positive control. These plasmids were introduced into the wild-type B128 strain and *ΔrhpA* mutant, and the strains were incubated at either 37°C or 33°C. The RhpA protein of strain B128 presents 98% amino acid identity with RhpA of X47-2AL.

In the presence of IPTG, the plasmid expressing *rhpA* fully restored growth of the *ΔrhpA* mutant at both temperatures. In contrast, neither the inducible copy of *rhlB* nor that of *csdA* restored growth of the Δ*rhpA* mutant at 33°C (Fig. 5C). Interestingly, expression of CsdA slightly improved growth of the Δ*rhpA* mutant at 37°C in liquid medium, suggesting partial complementation (the optical density [OD_600_] reached after 24 h of growth was twice that of the mutant with an empty vector) (Fig. S4).

Taken together, these results indicated that neither of these two *E. coli* helicases is able to fulfill the functionality of the *H. pylori* RhpA helicase.

10.1128/mBio.02071-17.4 FIG S4  *E. coli csdA* improves growth of the *H. pylori* B128 *ΔrhpA* mutant strain at 37°C but not at 33°C. Growth curves are for wild-type B128 and Δ*rhpA* mutant strains containing or not an IPTG-inducible copy of *E. coli csdA* cloned into vector pPH85. All strains were grown in the presence of 1 mM IPTG at 33°C and 37°C. Download FIG S4, DOCX file, 0.1 MB. Copyright © 2018 El Mortaji et al. 2018 El Mortaji et al. This content is distributed under the terms of the Creative Commons Attribution 4.0 International license.

### Analysis of the role of RhpA in ribosome biogenesis.

**FIG 6:**
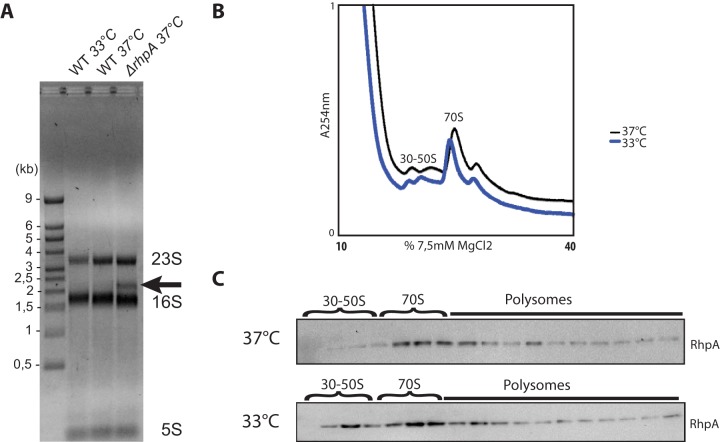
*H. pylori* grown at 33°C presents no visible defect in ribosome biogenesis but induces RhpA helicase recruitment to individual ribosomal subunits. (A) Total RNAs extracted from wild-type strain B128 grown at 33°C and 37°C and from the *ΔrhpA* mutant strain grown at 37°C were analyzed on 1% agarose gels. Positions of 23S, 16S, and 5S rRNA are indicated on the right. An additional band, marked by an arrow, was seen with the *ΔrhpA* mutant strain and was most probably a defect in 16S maturation. (B) Ribosome and polysome profiles of the wild-type (WT) *H. pylori* B128 wild-type strain grown at 37°C and 33°C were analyzed by sedimentation in sucrose gradients. At both temperatures, the pattern of sedimentation in sucrose was identical. (C) Western blotting with anti-RhpA antibodies of the sucrose gradient collected fractions showed a redistribution of the RhpA pool to isolated ribosomal subunits at 33°C.

In several microorganisms, at least one DEAD-box helicase has been shown to play a crucial role in ribosome biogenesis (9, 10). Since *H. pylori* has only one such helicase, we suspected that it might play a major role in ribosome biogenesis. However, in a previous paper in which we described the minimal *H. pylori* RNA degradosome (4), we observed no visible modification of the ribosome and polysome sedimentation profiles in the B128 Δ*rhpA* mutant compared to those of the wild-type strain. Nevertheless, in light of the essentiality of the *H. pylori* helicase during growth at 33°C, we decided to investigate further the contribution of RhpA in ribosome biogenesis at both 37°C and a low temperature (Fig. 6). First, we looked at the rRNA profiles by using total RNA extracted from a wild-type strain grown at 33°C or at 37°C and from a Δ*rhpA* mutant strain grown at 37°C (Fig. 6A). No visible changes were observed in the rRNA profiles of the wild-type strain grown at 37°C versus 33°C. Surprisingly, an additional band of 2.2 to 2.3 kb was reproducibly observed in the *ΔrhpA* mutant, suggesting a defect in 16S rRNA maturation in the absence of the helicase.

Second, we performed sucrose density gradient analysis with extracts from cultures of the wild-type strain that was grown at either 37°C or at 33°C. Under both conditions, similar sedimentation profiles were obtained (Fig. 6B). RhpA localization was analyzed by Western blotting on the collected fractions using anti-RhpA polyclonal antibodies as described in reference 4 (Fig. 6C). During growth at 37°C, RhpA is mainly associated with mature 70S ribosomes and polysomes, as we previously reported. At 33°C, RhpA is also associated with 70S ribosomes and polysomes, but the helicase is, in addition, detected in fractions corresponding to isolated 30S and 50S subunits, indicating a relocalization of an RhpA “pool” to these forms.

Taken together, these results suggest that RhpA is partially required for full maturation of 16S rRNA but that its absence does not result in a major ribosome biogenesis defect. In addition, at 33°C RhpA is not only associated with mature 70S ribosomes and polysomes (as found at 37°C) but it is also partially recruited to isolated ribosomal subunits. We conclude that RhpA plays a minor role in ribosome biogenesis with a more prominent function when *H. pylori* is exposed to lower temperatures.

### RhpA regulates the expression of the RNase J-encoding gene.

**FIG 7:**
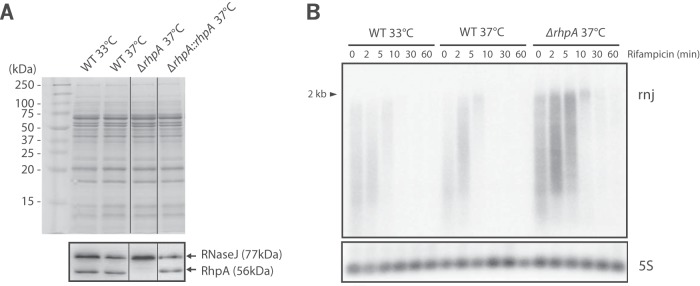
Expression and synthesis of RNase J, the major *H. pylori* ribonuclease, is under control of its degradosome partner, the RhpA helicase. (A) Western blotting of total extracts showed a similar increase in the level of RNase J in a B128 *ΔrhpA* mutant strain grown at 37°C and a wild-type strain grown at 33°C, compared to the wild-type (WT) strain grown at 37°C. RhpA levels were similar in all strains and under all conditions tested, except for the *ΔrhpA* mutant where it was absent, as expected. A Coomassie-stained SDS-PAGE gel served as a loading control. The RhpA bands of strains grown at 37°C were identical to those of the gel presented in Fig. 2. Spliced sites of the original gel are marked by a thin line. (B) Northern blot analysis of total RNA extracted from B128 wild-type cells grown at either 33°C or 37°C and from a *ΔrhpA* mutant strain grown at 37°C. Rifampin was added at time zero, and RNA was extracted at this time and the subsequent indicated time points (in minutes). Five micrograms of total RNA was separated on an agarose-formaldehyde gel and subsequently transferred on a nylon membrane prior to hybridization with RNA probes specific for *rnj* or 5S rRNA, which served as a loading control. A DNA marker was used for size estimation. The 2-kb full-length *rnj* transcript is shown by an arrow.

Since RhpA is a core component of the *H. pylori* RNA degradosome, we investigated whether it influences the expression of its partner, RNase J. For this aim, we first performed Western blotting on total extracts from the wild-type B128 and *ΔrhpA* mutant strains grown at 37°C (Fig. 7A). Using anti-RhpA-specific and anti-RNase J-specific antibodies (4), both RhpA and RNase J were found in similar amounts in the wild type and in the *rhpA* complemented strain. In contrast, significantly more (3.3-fold more) RNase J protein was detected in the *ΔrhpA* mutant. Given the major role of RhpA at low temperature, we also analyzed the amounts of both of the degradosome proteins during *H. pylori* growth at 33°C (Fig. 7A). At this temperature, no induction of RhpA protein was detected, showing that *rhpA* expression is not controlled by low temperature. In contrast, at 33°C, increased amounts of RNase J (3.5-fold more) were observed. Thus, the absence of RhpA and cold growth conditions result in enhanced RNase J production, suggesting a possible negative autoregulation of the *rnj* gene encoding RNase J that could possibly depend on a functional RNA degradosome. Feedback regulation has already been reported for *E. coli* RNase E, with *rne* transcripts being regulated by the activity of the RNA degradosome and the total RNase E protein level (28, 29). We therefore assessed the amounts of *rnj* transcripts in both wild-type and *ΔrhpA* mutant strains (Fig. 7B).

Northern blotting with an *rnj*-specific probe was performed on total RNA extracted at different time points after rifampin addition to the *H. pylori* culture (Fig. 7B). Reproducibly, only faint *rnj* transcripts were detected with RNA extracted from the wild-type strain, suggesting very fast decay under these conditions. In contrast, a much stronger *rnj* transcript signal was visible in the *ΔrhpA* mutant, although the signal also had a short half-life that was difficult to estimate. This indicated that the *rnj* transcript is more abundant and/or stabilized in the absence of RhpA. This *rnj* transcript reproducibly migrated as an RNA that is undergoing degradation; this was not due to experimental problems, since other RNAs of the same preparations migrated as distinct bands. In addition, a 2-kb *rnj* band was observed at 5 to 10 min after rifampin addition. This 2-kb band corresponds to the transcription unit of *rnj*, suggesting stabilization of the transcript by a mechanism that is yet to be elucidated. Finally, during growth at 33°C, we observed no major difference in the amounts of *rnj* transcripts.

**FIG 8:**
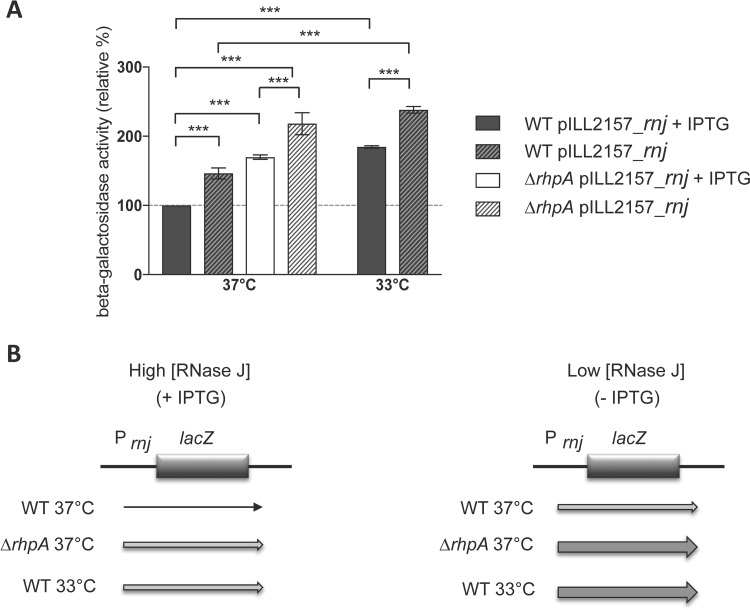
RhpA regulates the activity of the RNase J gene (*rnj*) promoter region. (A) β-Galactosidase activity expressed by the P*rnj*::*lacZ* reporter fusion inserted at the native locus of the B128 wild-type (WT) strain and its isogenic *ΔrhpA* mutant (both strains harbor pILL2157-*rnj*, expressing RNase J under control of an IPTG-inducible promoter). Cultures were incubated either at 37°C or during 5 h at a temperature of 33°C in the presence or absence of IPTG. β-Galactosidase activities are presented as ratios (percentages) of the activity measured under all conditions tested versus the reference wild-type strain, grown in the presence of 1 mM IPTG at 37°C. The wild-type strain displayed β-galactosidase activity of 533.8 (±3.34) Miller units. Each measurement corresponds to the mean value of duplicates from three independent experiments. Error bars represent standard deviations. ***, the mean value was significantly different between the two indicated strains compared (*P* < 0.001). (B) Schematic representation of the results. The thickness of the arrows is proportional to the expression level of the fusions. “High [RNase J]” data correspond to conditions with IPTG addition, and “low [RNase J]” data correspond to conditions without IPTG. The level of expression of the fusion increased (i) in the *ΔrhpA* context, (ii) when the wild-type bacteria were incubated at 33°C, and (iii) when RNase J was depleted by removal of IPTG from the culture medium.

In order to determine whether increased amounts of *rnj* transcripts in the *ΔrhpA* context are a reflection of stronger transcription or of diminished degradation, a transcriptional fusion between the *rnj* 5′-untranslated region (UTR) and *lacZ* was introduced at the native *rnj* chromosomal locus of a wild-type and a *ΔrhpA* strain. Since *rnj* is essential in *H. pylori*, these strains carried, in addition, a plasmid expressing an IPTG-inducible copy of *rnj* (4). In the presence of IPTG, the wild-type strain presented β-galactosidase activity of 533.8 (±3.34) Miller units. When these cells were shifted from 37°C to 33°C and grown for 5 h, the β-galactosidase activity was 1.8-fold higher, reflecting stronger activity of the *rnj* promoter region at the lower temperature (Fig. 8). In addition, the *ΔrhpA* mutant presented 1.7-fold-increased *rnj* promoter region activity. Finally, whatever the strain or the temperature, 5 h of growth without IPTG (which we previously showed caused depletion of RNase J protein [4]) generated a 1.3- to 1.4-fold increase in the activity of the *rnj* promoter region. Taken together, these results show that depletion of RNase J in the cell or the absence of RhpA leads to increased *rnj* promoter region activity. This is consistent with the existence of negative feedback autoregulation that acts on the 5′-UTR of *rnj* and depends on a functional RNA degradosome, whose activity relies on RNase J and on sufficient amounts of RhpA dedicated to the mRNA decay process.

## DISCUSSION

We investigated for the first time the role of the DEAD-box RNA helicase RhpA in the major human pathogen *H. pylori*. This study was motivated by two original characteristics of RhpA. First, it is one of the two components of a novel type of RNase J-based RNA degradosome that we discovered (4), and second, it is the sole protein of this family in *H. pylori*.

We first assessed the presence and possible conservation of the two protein partners of the RNA degradosome among the *Epsilonproteobacteria* class to which *H. pylori* belongs. Orthologous genes encoding RNase J were found in all analyzed *Epsilonproteobacteria* genomes. The tree obtained was well resolved except in the deepest branches and was congruent with the species tree. This suggested that the *rnj* genes were vertically transmitted from the last common ancestor of the *Epsilonproteobacteria* species. Phylogenetic analysis of the DEAD-box helicases distribution in the *Epsilonproteobacteria* class highlighted a striking feature: every member of the *Campylobacter* and *Helicobacter* genera, which included major pathogens such as *C. jejuni* and *H. pylori*, possesses either none or a sole DEAD-box helicase similar to RhpA. Accordingly, we predicted that RhpA was likely present in the common ancestor of the *Helicobacteraceae* family. RhpA is distantly related to the *E. coli* CsdA (cold shock protein) and, except for the DEAD-box motifs, this protein only presents low similarities with RhlB, the helicase of the *E. coli* RNA degradosome. Strikingly, acquisition of a distantly related DEAD-box helicase by only one member of the *Campylobacteraceae* family, *C. fetus* subsp. *fetus*, is likely to have occurred through horizontal gene transfer, possibly in *C. fetus* strains that colonize reptiles and have adapted to a host with fluctuating body temperature (30).

The free-living *Epsilonproteobacteria* that have larger genomes possess four to five DEAD-box helicases, except for the thermophile species *Nitratiruptor* sp., *Nautilia profundicola*, and *Nitratifractor salsuginis* genomes, which encode only one or three DEAD-box helicase genes. Therefore, the scarcity of DEAD-box helicases in the *Helicobacteraceae* and *Campylobacteraceae* families is probably not exclusively the result of their adaptation to less complex habitats followed by genome size reduction. Rather, we propose that it could be due to the restricted optimal growth temperature, in particular the propensity of some *Campylobacter* species to grow in hosts with a high body temperature, like birds.

Thus, the questions raised were whether in *H. pylori*, RhpA carries out the various functions described for the multiple helicases found in other organisms and how different the *H. pylori* RNA degradosome is from the one of *E. coli*.

DEAD-box helicases are involved in central cellular functions, such as ribosome biogenesis, RNA decay, and translation initiation (9, 10). In yeast, most DEAD-box helicases are essential for growth (31), while an *E. coli* mutant with the 5 DEAD-box helicase genes deleted is still viable (14). Deletion of *rhpA* in *H. pylori* has only a modest impact on growth at 37°C. It has been proposed that the nonessentiality of the DEAD-box helicases in bacteria is due to functional replacement by another enzyme with helicase activity. The best candidate is exoribonuclease RNase R. Indeed, in *E. coli* this enzyme has been shown to have RNA helicase activity *in vitro* (32). Moreover, RNase R is able to complement an *E. coli* growth defect at low temperature in the absence of CsdA (33). In *C. jejuni*, a major role of RNase R was found including impacts on bacterial growth and virulence traits, such as adhesion and invasion (34). An *H. pylori* RNase R mutant was viable and was reported to have enhanced motility and apoptosis induction (35). However, we did not confirm this motility phenotype for an *ΔrnaseR* mutant in the *H. pylori* X47-2AL background (36).

Cold sensitivity is a common phenotype of bacteria deficient for DEAD-box helicases, and often one of the corresponding genes is induced at low temperature (11). Growth of the *H. pylori ΔrhpA* mutant was totally inhibited at 33°C, a temperature only slightly lower than 37°C, its optimal growth temperature. This growth inhibition was reversible, since mutants recovered normal growth when shifted from 33°C to 37°C. In addition, synthesis of RhpA is not induced at 33°C. Cold sensitivity of helicase mutants has been explained by their inability to resolve one or more RNA secondary structures that are stabilized at lower temperatures, thereby blocking the expression of essential gene(s). Whether a 4°C shift in temperature is sufficient to produce such a dramatic effect on RNA structure stability is surprising and remains to be explored. Neither CsdA nor RhlB, DEAD-box helicases of the Gram-negative model organism *E. coli*, fully restored growth at 33°C to the *H. pylori ΔrhpA* mutant. This absence of functional complementation is probably related to differences between the *E. coli* and *H. pylori* partners of the helicases and to the fact that individual specialized helicases of *E. coli* cannot restore the activity of the “multifunctional” RhpA DEAD-box helicase in *H. pylori*.

We then searched for targets of RhpA and asked whether this protein plays a role in *H. pylori* virulence, as was reported for helicases found in *S. aureus* and *L. monocytogenes* (17, 18). We showed that RhpA is essential for *H. pylori* pathogenicity, as a *ΔrhpA* mutant was unable to colonize the stomach of a mouse model. Diminished motility of the *ΔrhpA* mutant, which we observed at 37°C, probably contributes to its loss of colonization capacity, but additional defects might also be involved. Motility has been previously shown to be essential for colonization by *H. pylori* (37). In *L. monocytogenes*, the motility defect of the helicase mutant was only observed at low temperature and was associated with diminished flagellin production (12). The *H. pylori ΔrhpA* mutant expresses wild-type amounts of the major flagellin FlaA and displays intact flagella, as demonstrated with electron microscopy. Assembly of functional motile flagella is a complex and highly regulated process (38, 39). *H. pylori* has a reduced number of transcriptional regulators, and we and others have revealed the importance of post-transcriptional control in this organism (4, 8, 40, 41). Therefore, we propose that RhpA controls the expression of a gene involved in energizing or the functioning of the flagellar machinery or in chemotaxis.

Urease is a major virulence factor of *H. pylori*. We previously showed that decay of the urease accessory gene mRNAs is controlled by RNase J (4, 8). We found that neither urease activity nor its expression was affected in the *ΔrhpA* mutant. This suggested, as we already proposed, that RNase J is active in two forms, alone and in association with RhpA. We are presently performing a comparative transcriptome analysis of the *ΔrhpA* mutant to define the individual and common targets of the two partners of the *H. pylori* RNA degradosome.

In bacteria with multiple DEAD-box helicases, one or more are required for proper ribosome biogenesis. In *E. coli*, the *csdA* mutants are affected in formation of mature 40S ribosomal particles (42). In *B. subtilis* and *L. monocytogenes*, helicase mutants present differences in the relative amounts of ribosome particles and mature ribosomes (15, 16). Polysome profiles were analyzed after fractionation of *H. pylori* extracts on a sucrose density gradient (as described in reference 4). No detectable differences in polysome profiles were observed in the *ΔrhpA* mutant when grown at 37°C or when the wild-type strain was grown at 33°C. In *L. monocytogenes*, increased levels of premature 23S rRNA were observed in several strains with mutations in the 4 DEAD-box helicases (15). In our work, analysis of rRNA revealed that a nonmature RNA species of approximately 2.2 to 2.3 kb accumulates in the *ΔrhpA* mutant, but not at 33°C. This species probably results from defective 16S rRNA maturation. In *H. pylori*, the enzymes involved in 16S rRNA maturation have not yet been defined. We previously showed that a mutant depleted for RNase J does not accumulate significant amounts of premature rRNAs and, in contrast with *B. subtilis*, the 5′-end maturation of the 16S rRNA is not affected (4, 8). Thus, the role of RhpA in rRNA maturation is probably not associated with the RNase J-based RNA degradosome but rather with a combined activity with another RNase yet to be identified. We previously demonstrated that RhpA is associated with translating ribosomes (70S and polysomes) (4). During growth at 33°C, we revealed a redistribution of the RhpA protein pool to individual ribosome subunits (30S and 50S). This raises the possibility that RhpA might be titrated by occluding secondary structures of rRNAs that are stabilized at lower temperatures, and under these conditions RhpA plays a role in the final steps of ribosomal biogenesis.

Finally, the RhpA mutant produced more RNase J protein and the corresponding mRNA was more abundant, although its half-life seemed similar to that of the parental wild-type strain. In addition, the activity of the 5′-UTR promoter region of the *rnj* gene was enhanced in the absence of either RhpA or RNase J. This strongly suggests that, in *H. pylori*, an active RNA degradosome composed of its two partners, RNase J and RhpA, controls the expression of RNase J by a feedback mechanism. This negative autoregulation of RNase J probably relies on, as in the case of RNase E (43), the 5′-untranslated promoter region of the *rnj* gene. At 33°C, the total amounts of RhpA in the cell are unchanged but a pool of RhpA is recruited to individual ribosomal subunits, suggesting that less protein might be available for the RNA degradosome. This could explain the enhanced amounts of RNase J protein produced at this lower temperature. We postulate that the RNase J negative feedback regulation allows this RNase to precisely adjust its synthesis to that of its substrates, one of them being its own mRNA.

In conclusion, we have shown that RhpA, the sole DEAD-box helicase of *H. pylori*, is essential for virulence. This multifunctional enzyme encompassing the various roles of the specialized DEAD-box helicases studied so far and the lack of redundancy certainly reflect the restricted niche of *H. pylori*. Further investigations will aim at a better understanding of the precise targets of the *H. pylori* minimal RNA degradosome and what precisely directs the selectivity of such a molecular machine.

## MATERIALS AND METHODS

### Ethics statement.

Experiments in mice were carried out in strict accordance with the recommendations in the Specific Guide for the Care and the Use of Laboratory Animals of the Institut Pasteur, according to the European Directive (2010/63/UE) and the corresponding French law on animal experimentation (Arrêtés de 1988). The protocol has been approved by the Committee of Central Animal Facility Board of the Institut Pasteur. To follow the new European directives, the project was approved by the CETEA, Comité d’éthique en Expérimentation Animale of the Institut Pasteur (2013-0051) and by the Ministère de l’Enseignement Supérieur et de la Recherche (751501).

### Bacterial strains and growth conditions.

The *H. pylori* strains used in this study (Table S1) are derivatives from X47-2AL (21, 22), 26695 (23), and B128 (24). Plasmids (Table S2) used to create or complement mutants of *H. pylori* were constructed and amplified using *E. coli* One Shot Top10 or DH5α strains (Thermo Fisher). *H. pylori* strains were grown on blood agar base 2 (Oxoid) plates supplemented with 10% defibrinated horse blood and with the following antibiotic-antifungal cocktail: amphotericin B at 2.5 μg/ml, polymyxin B 0.31 at μg/ml, trimethoprim at 6.25 μg/ml, and vancomycin at 12.5 μg/ml. Selection of *H. pylori* mutants was performed using kanamycin at 20 μg/ml, apramycin at 10 μg/ml, or chloramphenicol at 5 μg/ml. For liquid cultures, we used BHI broth (Oxoid) supplemented with 10% fetal calf serum (FCS; Eurobio), the antibiotic-antifungal cocktail, and the selective antibiotic when necessary. Motility assays were performed in 0.35% agar brucella broth medium (Oxoïd) complemented with 10% decomplemented FCS and antibiotic-antifungal cocktail and examined after 4 days. *H. pylori* cells were incubated at 33°C or 37°C under microaerophilic atmosphere conditions (6% O_2_, 10% CO_2_, 84% N_2_) using an Anoxomat atmosphere generator (MART Microbiology).

10.1128/mBio.02071-17.5 TABLE S1  Strain list. Download TABLE S1, DOCX file, 0.02 MB. Copyright © 2018 El Mortaji et al. 2018 El Mortaji et al. This content is distributed under the terms of the Creative Commons Attribution 4.0 International license.

10.1128/mBio.02071-17.6 TABLE S2  Plasmid list. Download TABLE S2, DOCX file, 0.02 MB. Copyright © 2018 El Mortaji et al. 2018 El Mortaji et al. This content is distributed under the terms of the Creative Commons Attribution 4.0 International license.

### Molecular techniques.

Molecular biology experiments were performed according to standard procedures (44) and the supplier’s (Fermentas) recommendations. The NucleoBond Xtra Midi kit (Macherey-Nagel) and QIAamp DNA minikit (Qiagen) were used for plasmid preparations and *H. pylori* genomic DNA extractions, respectively. PCRs were performed with either *Taq* core DNA polymerase (MP Biomedicals) or with Phusion Hot Start DNA polymerase (Finnzymes) when the product required a high-fidelity polymerase. The PCR8/GW/TOPO TA cloning kit (Invitrogen) was used to construct in *E. coli* the suicide plasmid that served for complementation in *H. pylori*.

### Construction of *H. pylori* mutant strains.

Chromosomal deletion of the entire gene encoding RhpA was performed in strains X47-2AL, 26695, and B128 (as described in reference 45). Briefly, fragments of about 600 bp upstream and downstream of the target gene were amplified by PCR (primers are listed in Table S3) and spliced into a nonpolar kanamycin or apramycin resistance cassette by overlap extension PCR. Deletions were introduced by allelic exchange. To complement the Δ*rhpA* mutation with a wild-type *rhpA* copy in *cis*, we first constructed a derivative of PCR8/GW/TOPO (suicide plasmid in *H. pylori*) carrying a 600-bp-long PCR fragment upstream from *rhpA* that was followed by three consecutive PCR fragments: (i) the *rhpA* wild-type gene, (ii) an apramycin resistance cassette, and (iii) a 600-bp-long fragment downstream from *rhpA*. Both *H. pylori* mutants were obtained by natural transformation as described previously (46) and selection on plates carrying the corresponding antibiotics.

10.1128/mBio.02071-17.7 TABLE S3  Primer list. Download TABLE S3, DOCX file, 0.02 MB. Copyright © 2018 El Mortaji et al. 2018 El Mortaji et al. This content is distributed under the terms of the Creative Commons Attribution 4.0 International license.

The *rhpA*, *rhlB*, and *csdA* genes were PCR amplified from the *H. pylori* X47-2AL or *E. coli* DH5α chromosomes by using the primer pairs indicated in Table S3 and were cloned into the pILL2157–*E. coli*-*H. pylori* shuttle vector and expressed under control of an IPTG-inducible promoter (47). To construct the P*rnj-lacZ-Kan* fusion, the *lacZ* and kanamycin resistance genes were PCR amplified using the primers described in Table S3, fused to the *rnj* promoter region, and introduced at the *rnj* native locus in a strain that carries a copy of *rnj* on a plasmid under control of an IPTG-inducible promoter (4). Deletion of the genes of interest, complementation on the chromosome or plasmids, and also correct insertion of cassettes were verified by PCR and sequencing of the corresponding genetic regions.

### Mouse model of colonization.

Seven NMRI specific-pathogen-free mice (Charles River Laboratories, Inc.) were orogastrically inoculated with 10^9^ CFU of the wild-type X47-2AL parental strain, the Δ*rhpA* mutant, or the complemented strain prepared in 100 μl of peptone broth. As described in reference 48, 1 month after inoculation, mice were sacrificed and stomachs were crushed in peptone broth. Viable *H. pylori* bacteria colonizing the stomach were enumerated by culture of serial dilutions of homogenized tissue on blood agar plates containing, in addition, bacitracin (200 μg/ml) and nalidixic acid (10 μg/ml).

### Scanning electron microscopy.

Exponentially growing bacteria were fixed in a buffer containing 2.5% glutaraldehyde in 0.1 M cacodylate (pH 7.2). Samples were dehydrated through a graded series of 25, 50, 75, and 95% ethanol solution and then dehydrated in 100% ethanol, followed by critical point drying with CO_2_. Dried samples were sputter coated with 10-nm gold palladium particles by using a Gatan ion beam coater and were examined and photographed with a JEOL JSM 6700F field emission scanning electron microscope operating at 5 kV. Images were acquired with the upper SE detector (SEI).

### Western blotting.

*H. pylori* cells were grown to an OD_600_ of 0.8, harvested by centrifugation, and then washed twice in phosphate-buffered saline (PBS) prior to be disruption by sonication in a lysis buffer containing 10 mM Tris-HCl (pH 7.5), 5 mM β-mercaptoethanol, and complete protease inhibitor cocktail (Roche). Cell debris was removed by centrifugation, and supernatants were collected as total extracts. Protein amounts in each sample were calibrated using the Bradford DC protein assay (Bio-Rad) with bovine serum albumin (BSA) as a standard. Twenty micrograms of proteins was loaded and separated on a 4-to-20% Mini-Protean TGX precast protein gel (BioRad) and subsequently electrotransferred on a polyvinylidene difluoride (PVDF) membrane (Bio-Rad). The *H. pylori* RhpA, RNase J, UreA, UreB, and FlaA proteins were detected with rabbit polyclonal antibodies anti-RhpA and anti-RNase J (4), anti-UreA and anti-UreB (49), and anti-FlaA (50) at the respective dilutions of 1:5,000, 1:700, 1:500, 1:5,000, and 1:2,000. Goat anti-rabbit IgG–horseradish peroxidase conjugate (Santa Cruz Biotechology) was used as secondary antibody at a 1:10,000 dilution, and the detection was achieved with chemiluminescent substrate ECL reagent (Pierce).

### Ribosome profiling on sucrose gradient.

Aliquots of 250 μl of *H. pylori* cells from cultures grown at 33°C or 37°C to an OD_600_ of 0.6 were incubated at 4°C during 10 min with 100 mg/ml chloramphenicol to avoid polysome runoff. The preparation of samples, sucrose gradients, and fraction collection were performed as described in reference 4. Proteins of the different fractions were precipitated with trichloroacetic acid and loaded on 10% acrylamide gels for Western blotting using anti-RhpA specific antibodies (4). After protein transfer, amido black staining of the membranes was employed to verify that the samples loaded were comparable for each growth condition tested.

### Total RNA extraction and Northern blotting.

Total RNA was extracted at different time points after rifampin addition (80 µg/ml) from 5-ml samples of *H. pylori* cultures at an OD_600_ of 0.5 to 0.9 by using a NucleoSpin miRNA kit from Macherey-Nagel (4). For Northern blotting, 5 µg of RNA was separated on an agarose-formaldehyde gel and transferred to a Hybond N+ membrane (Amersham Biosciences, Inc.) overnight by passive transfer in 10× SSC (pH 7) buffer (1× SSC is 0.15 M NaCl plus 0.015 M sodium citrate). Transferred RNA was fixed to the membrane via UV irradiation for 2 min. The membrane was blocked for 45 min at 65°C with ULTRAhyb hybridization buffer (Ambion), then 5 µl of ^32^P-labeled riboprobe was added and the membrane was further incubated overnight at the same temperature. After three washes for 10 min at 65°C with 2× SSC, 0.2% SDS, the membrane was exposed to a phosphorimager screen (Kodak) and scanned with an FLA-9000 phosphorimager (Fujifilm).

### β-Galactosidase activity assay.

Briefly, cultures of B128 *Δrnj*::*Prnj-lacZ-Kan*(pILL2157-*rnj*) and B128 *Δrnj*::*Prnj-lacZ-Kan ΔrhpA*::*apra*(pILL2157-*rnj*) strains were grown in BHI medium supplemented with 10% FCS and 1 mM IPTG until an OD_600_ of 0.3 was reached. Then, 0.5-ml samples of these cultures were subjected to the β-galactosidase assay, and provided the reference value for β-galactosidase activity (51). Remaining bacterial cultures were either grown at 37°C, shifted to 33°C, and/or washed and subsequently incubated in medium without IPTG at both temperatures. After 5 h, 0.5-ml samples were washed twice with 1× phosphate-buffered saline and further permeabilized with 100 µl chloroform and 50 µl of 0.1% SDS in Z buffer containing 70 mM Na_2_HPO_4_ ⋅ 12H_2_O, 30 mM NaHPO_4_ ⋅ H_2_O, 1 mM MgSO_4_, and 0.2 mM MnSO_4_. Samples were vortexed briefly and incubated at 28°C for 2 min. The enzymatic reaction was started by adding 0.5 ml *o*-nitrophenyl-β-d-galactopyranoside at 4 mg/ml and stopped by addition of 0.5 ml 1 M Na_2_CO_3_ when sufficient yellow color was reached. The percentage of β-galactosidase activity was calculated as described elsewhere (52) from 3 independent experiments performed in duplicates. Results are presented as percentages relative to the control activity obtained for the reference strain grown at 37°C in the presence of IPTG.

### Phylogenomics analyses. (i) Data sample.

For our phylogenomics analyses, the complete genomes of the *Epsilonproteobacteria* were retrieved from the EBI database (https://www.ebi.ac.uk/genomes/bacteria.html). A total of 28 species were used in this analysis (Table S4). An RPS-BLAST database was constructed with the NCBI CDDS database. We used the COG0513 profile to build the list of DEAD-box helicase candidate proteins (Table S5). Three DEAD-box helicase partial sequences were found in the N-terminal region. Analysis of the DNA sequences of the genomes, with tblastn and a full-length DEAD-box helicase sequence as the query, revealed that they corresponded to annotation errors. The revised sequences were thus used for further analysis.

10.1128/mBio.02071-17.8 TABLE S4  *Epsilonproteobacteria* species used. Download TABLE S4, DOCX file, 0.02 MB. Copyright © 2018 El Mortaji et al. 2018 El Mortaji et al. This content is distributed under the terms of the Creative Commons Attribution 4.0 International license.

10.1128/mBio.02071-17.9 TABLE S5  DEAD-box helicase proteins. Download TABLE S5, DOCX file, 0.04 MB. Copyright © 2018 El Mortaji et al. 2018 El Mortaji et al. This content is distributed under the terms of the Creative Commons Attribution 4.0 International license.

### (ii) Protein trees.

The sequences were aligned with the program mafft. The alignment was trimmed with trimAl with the -automated1 option, which is optimized for maximum likelihood tree reconstruction programs (53). We used the ProtTest3 program (54) to select the optimal combination of parameters. The LG model of sequence evolution, with a γ-correction (four categories of evolutionary rates) and shape parameter and proportion of invariant sites estimated from data, was retained. The trees were computed with PhyML (55). The tree space was explored with subtree pruning and regrafting (SPR) topological moves. The statistical branch support was inferred with nonparametric bootstrap sampling (100 samples). The tree was drawn with iTOL (56). The same procedure was applied for the identification of RNase J homologues (Table S6).

10.1128/mBio.02071-17.10 TABLE S6  RNase J proteins. Download TABLE S6, DOCX file, 0.02 MB. Copyright © 2018 El Mortaji et al. 2018 El Mortaji et al. This content is distributed under the terms of the Creative Commons Attribution 4.0 International license.

### (iii) Species tree.

The phylogenetic tree was based on the concatenation of alignments obtained for a set of conserved families of protein sequences. A set of COG families that included one sequence of each strain without paralogues was retained. A multiple alignment of each family was performed with mafft. The quality of the alignments was estimated with trimAL. Only alignments with few gaps and high site conservations were used to compute the tree. The ProtTest3 program (54) was used on each alignment. The LG model of sequence evolution with a γ-correction (eight categories of evolutionary rates) and shape parameter and proportion of invariant sites estimated from data were retained. The aligned sequences of 154 COG families for the 28 species were concatenated to produce a single alignment of 55,217 aligned positions, including 14,704 sites without polymorphism (26.63%). The tree was computed with PhyML (55) and rooted according to methods described in reference 57. Our tree topology was fully congruent with the one published by those authors, and all internal nodes were supported by a bootstrap value of 100%. The tree was drawn with iTOL (56).
