# Supplementary figures and images for: The Sole DEAD-Box RNA Helicase of the Gastric Pathogen Helicobacter pylori Is Essential for Colonization
Source: mBio. 2018 Mar 27;9(2):e02071-17. doi: 10.1128/mBio.02071-17 (PMC5874925; doi:10.1128/mBio.02071-17)

**Supplementary figures**

**Figure S1: Phylogenetic tree of RNase J from Epsilonproteobacteria**

**
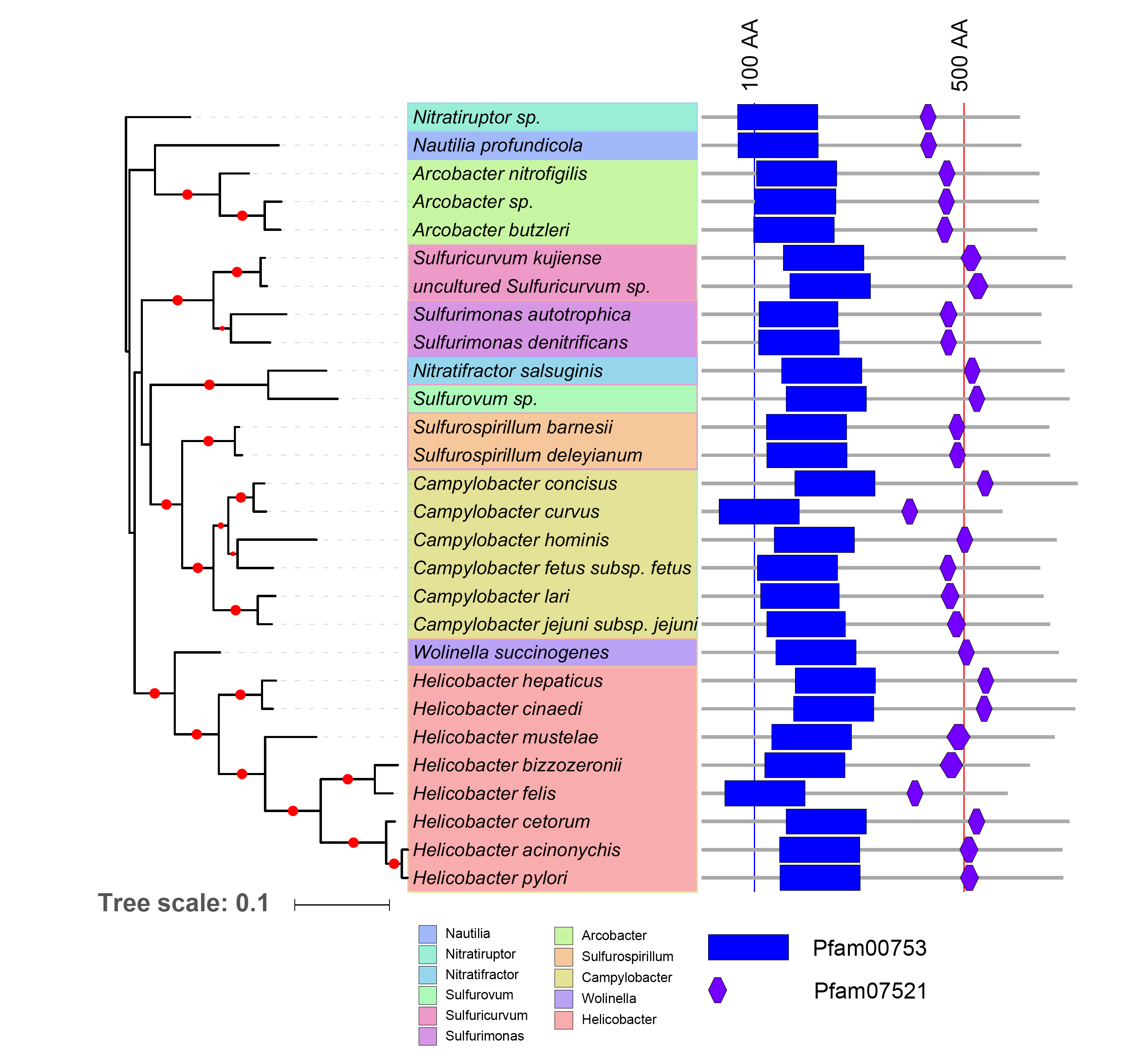
**

Supplement: FIG S1 [file mbo001183784sf1.docx]
