# Supplementary material for: The Sole DEAD-Box RNA Helicase of the Gastric Pathogen Helicobacter pylori Is Essential for Colonization
Source: mBio. 2018 Mar 27;9(2):e02071-17. doi: 10.1128/mBio.02071-17 (PMC5874925; doi:10.1128/mBio.02071-17)
Supplement: FIG S2 [file mbo001183784sf2.docx]

**Supplementary figures**

**Figure S2: Phylogenetic tree of DEAD-box helicases from Epsilonproteobacteria**
