# Supplementary material for: The Sole DEAD-Box RNA Helicase of the Gastric Pathogen Helicobacter pylori Is Essential for Colonization
Source: mBio. 2018 Mar 27;9(2):e02071-17. doi: 10.1128/mBio.02071-17 (PMC5874925; doi:10.1128/mBio.02071-17)
Supplement: FIG S3 [file mbo001183784sf3.docx]

**Supplementary figures**

**Figure S3**: **Synthesis of the urease structural subunits and urease activity are not controlled by RhpA (strain X47-2AL).**

**A B**
