# Supplementary material for: The Sole DEAD-Box RNA Helicase of the Gastric Pathogen Helicobacter pylori Is Essential for Colonization
Source: mBio. 2018 Mar 27;9(2):e02071-17. doi: 10.1128/mBio.02071-17 (PMC5874925; doi:10.1128/mBio.02071-17)
Supplement: FIG S4 [file mbo001183784sf4.docx]

**Supplementary figures**

**Figure S4**: ***E. coli csdA* improves growth of the *H. pylori* B128 *∆rhpA* mutant strain at 37°C but does not restore growth at 33°C**.
