# Supplementary material for: The Sole DEAD-Box RNA Helicase of the Gastric Pathogen Helicobacter pylori Is Essential for Colonization
Source: mBio. 2018 Mar 27;9(2):e02071-17. doi: 10.1128/mBio.02071-17 (PMC5874925; doi:10.1128/mBio.02071-17)
Supplement: TABLE S1 [file mbo001183784st1.docx]

**Supplementary material:**

Table S1: Strain List

| **Name** | **Description** | **Plasmid** | **Resistance (*)** | **Reference** |
| --- | --- | --- | --- | --- |
| X47-2AL | Wild-type strain | none | - | (1, 2) |
| X47-2AL*ΔrhpA* | *rhpA ::aphA-3* | none | Kan | This study |
| X47-2AL*ΔrhpA ::rhpA* | *aphA-3 ::rhpA-aac(3)-IV* | none | Apr | This study |
| B128 | Wild-type strain | none | - | (3) |
| B128 + pPH85 | Wild-type strain | pPH85 | Cm | This study |
| B128 + pPH85_*rhpA* | Wild-type strain | pPH85_*rhpA* | Cm | This study |
| B128 + pPH85_*rhlB* | Wild-type strain | pPH85_*rhlB* | Cm | This study |
| B128 + pPH85_*csdA* | Wild-type strain | pPH85_*csdA* | Cm | This study |
| B128*ΔrhpA* | *rhpA ::aphA-3* | none | Kan | This study |
| B128*ΔrhpA* + pPH85 | *rhpA ::aphA-3* | pPH85 | Kan, Cm | This study |
| B128*ΔrhpA* + pPH85_*rhpA* | *rhpA ::aphA-3* | pPH85_*rhpA* | Kan, Cm | This study |
| B128*ΔrhpA* + pPH85_*rhlB* | *rhpA ::aphA-3* | pPH85_*rhlB* | Kan, Cm | This study |
| B128*ΔrhpA* + pPH85_*csdA* | *rhpA ::aphA-3* | pPH85_*csdA* | Kan, Cm | This study |
| B128∆*rnj::Prnj-lacZ-Kan* + pILL2157-*rnj* | *∆rnj::Prnj-lacZ-Kan* | pILL2157_*rnj* | Kan, Cm | This study |
| B128*∆rnj::Prnj-lacZ-Kan ∆rhpA::Apra* + pILL2157-*rnj* | *∆rnj::Prnj-lacZ-Kan ∆rhpA::Apra* | pILL2157_*rnj* | Kan, Cm | This study |

(*) Kan : Kanamycin ; Apr : Apramycin ; Cm : Chloramphenicol

References

1. Ermak TH, Giannasca PJ, Nichols R, Myers GA, Nedrud J, Weltzin R, Lee CK, Kleanthous H, Monath TP. 1998. Immunization of mice with urease vaccine affords protection against *Helicobacter pylori* infection in the absence of antibodies and is mediated by MHC class II-restricted response. J Exp Med 188:2277-2288.

2. Veyrier FJ, Ecobichon C, Boneca IG. 2013. Draft Genome Sequence of Strain X47-2AL, a Feline *Helicobacter pylori* Isolate. Genome Announcements 1:e01095-13.

3. McClain MS, Shaffer CL, Israel DA, Peek RMJ, Cover TL. 2009. Genome sequence analysis of *Helicobacter pylori* strains associated with gastric ulceration and gastric cancer. BMC Genom 10:3:doi: 10.1186/1471-2164-10-3.
