# Supplementary material for: The Sole DEAD-Box RNA Helicase of the Gastric Pathogen Helicobacter pylori Is Essential for Colonization
Source: mBio. 2018 Mar 27;9(2):e02071-17. doi: 10.1128/mBio.02071-17 (PMC5874925; doi:10.1128/mBio.02071-17)
Supplement: TABLE S2 [file mbo001183784st2.docx]

**Supplementary material:**

Table S2 : Plasmid list

| **Name** | **Description** | **Resistance (*)** | **Reference** |
| --- | --- | --- | --- |
| pILL2157 | Derivative of the pHeL2 *E. coli*-*H. pylori* shuttle vector –carries *lacZ* controlled by p*ureI* with 2 LacI-binding sites in the MCS | Cm | (4) |
| pPH85 | pILL2157 without *lacZ* | Cm | This study |
| pPH85_*rhpA* | *rhpA* locus from *H. pylori* B128 cloned into pPH85 | Cm | This study |
| pPH85_*rhlB* | *rhlB* gene from *E. coli* DH5α cloned into pPH85 | Cm | This study |
| pPH85_*csdA* | *csdA* gene of *E. coli* DH5α cloned into pPH85 | Cm | This study |
| pPH074 | pCR8/GW/TOPO (invitrogen) - HP0274-aac(3)-IV cloned into TOPO sites. Suicide plasmid for *rhpA* recomplementation | Spec | This study |
| pILL2157_*rnj* | *rnj* locus cloned into pILL2157 | Cm | (5) |

(*) Cm : Chloramphenicol ; Spec: Spectinomycin

References

4. Boneca IG, De Reuse H, Epinat J-C, Pupin M, Labigne A, Moszer I. 2003. A revised annotation and comparative analysis of *Helicobacter pylori* genomes. Nucl Acid Res 31:1704-1714.

5. Redko Y, Galtier E, Arnion H, Darfeuille F, Sismeiro O, Coppée J-Y, Médigue C, Weiman M, Cruveiller S, De Reuse H. 2016. RNase J depletion leads to massive changes in mRNA abundance in *Helicobacter pylori.* RNA Biol 13:243-253.
