# Supplementary material for: The Sole DEAD-Box RNA Helicase of the Gastric Pathogen Helicobacter pylori Is Essential for Colonization
Source: mBio. 2018 Mar 27;9(2):e02071-17. doi: 10.1128/mBio.02071-17 (PMC5874925; doi:10.1128/mBio.02071-17)
Supplement: TABLE S3 [file mbo001183784st3.docx]

**Supplementary material:**

Table S3 : Primer list

| **Name** | **Sequence 5’🡪3’** | **Description** |
| --- | --- | --- |
|  |  |  |
| **oUPH1029** | GGTAAGGAGGCTGTAATCATCAC | ***rhpA* deletion :**Forward primer to amplify upstream region of *HP0247* (*rhpA*) |
| **oUPH1030** | GTTAGTCACCCGGGTACCTTTATAAAAAGCTAATAAAAGGC | ***rhpA* deletion :** Reverse primer to amplify upstream region of *HP0247* (*rhpA*). Primer contains a homolougous region to the Kanamycin resistance cassette |
| **oUPH1031** | TACCTGGAGGGAATAAATATTTAAAAAGGAAATTCATGCCC | ***rhpA* deletion :** Forward primer to amplify downstream region of *HP0247* (*rhpA*). Primer contains a homolougous region to the Kanamycin resistance cassette |
| **oUPH1032** | GCGCACCACAGGGTTGATG | ***rhpA* deletion :** Reverse primer to amplify upstream region of *HP0247* (*rhpA*). |
| **oUPH832** | CGGTACCCGGGTGACTAA | ***rhpA* deletion :** Forward primer to amplify *aphaA-3* (Kanamycin) resistance cassette. |
| **oUPH918** | ACTCTAGAGGATCCCCGGGT | ***rhpA* deletion :** Reverse primer to amplify *aphaA-3* (Kanamycin) resistance cassette. |
| **oUPH884** | ATGGAATTGAATCAACCACCACTCC | **pPH074 construction :** Forward primer to amplify *HP0247* (*rhpA*) gene. |
| **oUPH885** | TTAACGGCGTTTGGGTTTTTTAGAATA | **pPH074 construction :** Reverse primer to amplify *HP0247* (*rhpA*) gene. |
| **oUPH886** | AATATTTAAAAAGGAAATTCATGCCC | **pPH074 construction :**Forward primer to amplify *HP0248*. |
| **oUPH887** | GCGCACCACAGGGTTGATG | **pPH074 construction :** Reverse primer to amplify *HP0248*. |
| **oUPH888** | TATTCTAAAAAACCCAAACGCCGTTAAGGGTTCATGTGCAGCTCCA | **pPH074 construction :** Forward primer to amplify *aac(3)-IV* (gentamycin) resistance cassette (contains an oUPH885 homologous region). |
| **oUPH889** | GGGCATGAATTTCCTTTTTAAATATTCGATCCGCTCCACGTGTTGCC | **pPH074 construction :** Reverse primer to amplify *aac(3)-IV* (gentamycin) resistance cassette (contains an oUPH886 homologous region). |
| **oEG_059** | GGATAACAATTTTGTTTGGAAGGAAAAGCAATGAACGTTTTTAATCCCGC | **pILL2157bis_csdA construction :** Forward primer to clone *E.coli csdA* locus into pILL2157bis (contains NdeI restriction site and pILL2157bis homologous region) |
| **oEG_060** | ATTTTCAACGTGGATCTGAATTCGAGCTCGGGGTTTGTCATTAATCCACC | **pILL2157bis_csdA construction :** Reverse primer to clone *E. coli csdA* locus into pILL2157bis (contains KpnI restriction site and pILL2157bis homologous region) |
| **oUPH1180** | AATAAACATATGAGCAAAACACATTTAACAGAAC | **pILL2157bis_rhlB construction :** Forward primer to clone *E. coli* *rhlB* locus into pILL2157bis (contains NdeI restriction site) |
| **oUPH1181** | AATATAAGGATCCTTAACCTGAACGACGACGATTACGC | **pILL2157bis_rhlB construction :** Reverse primer to clone *E. coli* *rhlB* locus into pILL2157bis (contains BamHI restriction site) |
| **oUPH1176** | AAATAACATATGGAATTGAATCAACCACCACTCC | **pILL2157bis_rhpA construction :** Forward primer to clone *H. pylori* *rhpA* locus into pILL2157bis (contains NdeI restriction site) |
| **oUPH1177** | ATTTAAGGATCCTTAACGGCGTTTGGGTTTTTTAGAATA | **pILL2157bis_rhpA construction :** Reverse primer to clone *H. pylori* *rhpA* locus into pILL2157bis (contains BamHI restriction site) |
| **oLEM222** | CGCTAAAGATTCACAGAAC | **LacZ fusion with *rnj* promoter :** Forward primer to amplify upstream region of *rnj* for transcriptionnal fusion of lacZ with *rnj* promoter |
| **oLEM223** | CGTAATCATGGTACTAGTcatAACGATTATTCCTTTATTTCAATTT | **LacZ fusion with *rnj* promoter :** Reverse primer to amplify upstream region of *rnj* for transcriptionnal fusion of lacZ-kan with *rnj* promoter. primer contains a complementary region to lacZ. |
| **oLEM047** | atgACTAGTACCATGATTACG | **LacZ fusion with *rnj* promoter**: Forward primer to amplify l*acZ* gene fused to the Kanamycin cassette. |
| **oLEM010** | CATTATTCCCTCCAGGTAC | **LacZ fusion with *rnj* promoter**: Reverse primer to amplify l*acZ* gene fused to the Kanamycin cassette. |
| **oLEM224** | gtacctggagggaataATGCCCATTCTTTTtgaTTGTAACG | **LacZ fusion with *rnj* promoter :** Forward primer to amplify downstream region of *rnj* for transcriptionnal fusion of lacZ-Kan with *rnj* promoter. Primer contains a complementary region to the kanamycin cassette. |
| **oLEM225** | CCATTAAAACATCGCCTAAC | **LacZ fusion with *rnj* promoter :** Reverse primer to amplify downstream region of *rnj* for transcriptionnal fusion of lacZ-Kan with *rnj* promoter. |
| **oLEM227** | CAAGGAGGCTGTAATCATC | ***rhpA* deletion :** Forward primer to amplify upstream region of *rhpA* in B128 |
| **oLEM228** | gtattgcacgaCATtgcactccGGGAGATTCATACCTCAA | ***rhpA* deletion :** Reverse primer to amplify upstream region of *rhpA* in B128. Primer contains a homologous region to the apramycin cassette. |
| **oLEM229** | ggctgatacctggagggaataatgCCCATTGATTTGAACG | ***rhpA* deletion :** Forward primer to amplify downstream region of *rhpA* in B128. Primer contains a homologous region to the apramycin cassette and a RBS sequence for downstream gene |
| **Name** | **Sequence 5’🡪3’** | **Description** |
|  |  |  |
| **oLEM230** | GCGCACCACAGGGTTGATG | ***rhpA* deletion**: Reverse primer to amplify downstream region of *rhpA* in B128 |
| **oLEM209** | CTAATACGACTCACTATACATTTCCCTATCCCTGCACCGACC | **Northern Blot** Forward primer to make riboprobe complementary to 5S. T7 promoter |
| **oLEM210** | AGAGAAGAGGAACTACCC | **Northern Blot :** Reverse primer to make riboprobe complementary to 5S (5S-FW) |
| **oLEM211** | CTAATACGACTCACTATAGGCGATGCTCTTTTTTATG | **Northern Blot :** Forward primer to make riboprobe complementary to rnj. T7 promoter |
| **oLEM212** | CTGATAACAACCATTATGAAAAC | **Northern Blot** Reverse primer to make riboprobe complementary to rnj |
| **oLEM215** | TCGGAATGGTTAACTGGGTAGTTCCT | **Northern Blot 5S** |
| **oLEM216** | CTTTCATTGTTTTCATAATGG | **Northern Blot rnj** |
|  |  |  |
