# Supplementary material for: The Sole DEAD-Box RNA Helicase of the Gastric Pathogen Helicobacter pylori Is Essential for Colonization
Source: mBio. 2018 Mar 27;9(2):e02071-17. doi: 10.1128/mBio.02071-17 (PMC5874925; doi:10.1128/mBio.02071-17)
Supplement: TABLE S4 [file mbo001183784st4.docx]

**Supplementary material:**

Table S4 : Epsilonproteobacteria species used

| **Species** | **Strain** | **Size (pb)** | **Proteome** | **Version** |
| --- | --- | --- | --- | --- |
| *Arcobacter butzleri* | RM4018 | 2341251 | 2259 | CP000361.1 |
| *Arcobacter nitrofigilis* | DSM 7299 | 3192235 | 3126 | CP001999.1 |
| *Arcobacter sp.* | L | 2945673 | 2845 | AP012048.1 |
| *Campylobacter concisus* | 13826 | 2052007 | 2024 | CP000792.1 |
| *Campylobacter curvus* | 525.92 | 1971264 | 1931 | CP000767.1 |
| *Campylobacter fetus* | 82-40 | 1773615 | 1719 | CP000487.1 |
| *Campylobacter hominis* | ATCC BAA-381 | 1711273 | 1682 | CP000776.1 |
| *Campylobacter jejuni* | NCTC 11168 | 1641481 | 1624 | AL111168.1 |
| *Campylobacter lari* | RM2100; ATCC BAA-1060D | 1525460 | 1504 | CP000932.1 |
| *Helicobacter acinonychis* | Sheeba | 1553927 | 1612 | AM260522.1 |
| *Helicobacter bizzozeronii* | CIII-1 | 1755458 | 1894 | FR871757.1 |
| *Helicobacter cetorum* | MIT 00-7128 | 1947646 | 1717 | CP003479.1 |
| *Helicobacter cinaedi* | PAGU611 | 2078348 | 2096 | AP012344.1 |
| *Helicobacter felis* | ATCC 49179 | 1672681 | 1654 | FQ670179.2 |
| *Helicobacter hepaticus* | ATCC 51449 | 1799146 | 1875 | AE017125.1 |
| *Helicobacter mustelae* | 12198 | 1578097 | 1400 | FN555004.1 |
| *Helicobacter pylori* | 26695 | 1667867 | 1566 | AE000511.1 |
| *Nitratiruptor sp.* | SB155-2 | 1877931 | 1843 | AP009178.1 |
| *Nautilia profundicola* | AmH | 1676444 | 1730 | CP001279.1 |
| *Nitratifractor salsuginis* | DSM 16511 | 2101285 | 2088 | CP002452.1 |
| *Sulfurimonas autotrophica* | DSM 16294 | 2153198 | 2158 | CP002205.1 |
| *Sulfurospirillum barnesii* | SES-3 | 2510109 | 2491 | CP003333.1 |
| *uncultured Sulfuricurvum sp.* | RIFRC-1 | 2358861 | 2358 | CP003920.1 |
| *Sulfurospirillum deleyianum* | DSM 6946 | 2306351 | 2265 | CP001816.1 |
| *Sulfuricurvum kujiense* | DSM 16994 | 2574824 | 2538 | CP002355.1 |
| *Sulfurovum sp.* | NBC37-1 | 2562277 | 2438 | AP009179.1 |
| *Sulfurimonas denitrificans* | DSM 1251 | 2201561 | 2096 | CP000153.1 |
| *Wolinella succinogenes* | DSMZ 1740 | 2110355 | 2044 | BX571656.1 |
