# Supplementary material for: The Sole DEAD-Box RNA Helicase of the Gastric Pathogen Helicobacter pylori Is Essential for Colonization
Source: mBio. 2018 Mar 27;9(2):e02071-17. doi: 10.1128/mBio.02071-17 (PMC5874925; doi:10.1128/mBio.02071-17)
Supplement: TABLE S5 [file mbo001183784st5.docx]

**Supplementary material:**

Table S5 : DEAD-box helicase proteins

| **Name** | **Family** | **Length** | **Function** | **SP link** | **Species** | **Strain** |
| --- | --- | --- | --- | --- | --- | --- |
| DBPA | DbpA | 453 | ATP-independent RNA helicase DbpA | [A6QC93](http://www.uniprot.org/uniprot/A6QC93) | *Sulfurovum sp.* | NBC37-1 |
| ADN10122.1 | DbpA | 452 | ATP-dependent RNA helicase DbpA | [E0URV8](http://www.uniprot.org/uniprot/E0URV8) | *Sulfurimonas autotrophica* | DSM 16294 |
| ABB43422.1 | DbpA | 457 | DEAD/DEAH box helicase-like protein | [Q30UA9](http://www.uniprot.org/uniprot/Q30UA9) | *Sulfurimonas denitrificans* | DSM 1251 |
| ADR33847.1 | DbpA | 450 | DEAD/DEAH box helicase domain protein | [E4TX06](http://www.uniprot.org/uniprot/E4TX06) | *Sulfuricurvum kujiense* | DSM 16994 |
| AFV97328.1 | DbpA | 450 | ATP-dependent RNA helicase DbpA | [K7S3U0](http://www.uniprot.org/uniprot/K7S3U0) | *uncultured Sulfuricurvum sp.* | RIFRC-1 |
| DBPA | DBPA | 457 | enzyme; RNA synthesis, modification, DNA transcriptionATP-dependent RNA helicase, specific for 23S rRNA | [P21693](http://www.uniprot.org/uniprot/P21693) | *Escherichia coli* | K-12 |
| AFL68118.1 | DbpA | 463 | DNA/RNA helicase, superfamily II | [I3XVZ4](http://www.uniprot.org/uniprot/I3XVZ4) | *Sulfurospirillum barnesii* | SES-3 |
| ACZ11783.1 | DbpA | 463 | DEAD/DEAH box helicase domain protein | [D1B113](http://www.uniprot.org/uniprot/D1B113) | *Sulfurospirillum deleyianum* | DSM 6946 |
| ADG92956.1 | DbpA | 452 | DEAD/DEAH box helicase domain protein | [D5V4Q1](http://www.uniprot.org/uniprot/D5V4Q1) | *Arcobacter nitrofigilis* | DSM 7299 |
| BAK73878.1 | DbpA | 455 | ATP-dependent RNA helicase | [G2HXM5](http://www.uniprot.org/uniprot/G2HXM5) | *Arcobacter sp.* | L |
| DBPA | DbpA | 454 | ATP-dependent RNA helicase DbpA | [A8EV19](http://www.uniprot.org/uniprot/A8EV19) | *Arcobacter butzleri* | RM4018 |
| DEAD | DEAD | 629 | enzyme; RNA synthesis, modification, DNA transcriptionATP-dependent RNA helicase | [P0A9P6](http://www.uniprot.org/uniprot/P0A9P6) | *Escherichia coli* | K-12 |
| ACM92187.1 | CsdA | 467 | ATP-dependent RNA helicase DeaD | [B9L6F6](http://www.uniprot.org/uniprot/B9L6F6) | *Nautilia profundicola* | AmH |
| DEAD | CsdA | 471 | ATP-dependent RNA helicase, DEAD-box family | [A6Q5Q6](http://www.uniprot.org/uniprot/A6Q5Q6) | *Nitratiruptor sp.* | SB155-2 |
| ADV46983.1 | CsdA | 477 | DEAD/DEAH box helicase domain protein | [E6X1C1](http://www.uniprot.org/uniprot/E6X1C1) | *Nitratifractor salsuginis* | DSM 16511 |
| DEAD | CsdA | 492 | ATP-dependent RNA helicase, DEAD-box family | [A6Q8Y9](http://www.uniprot.org/uniprot/A6Q8Y9) | *Sulfurovum sp.* | NBC37-1 |
| ADG91801.1 | CsdA | 488 | DEAD/DEAH box helicase domain protein | [D5V468](http://www.uniprot.org/uniprot/D5V468) | *Arcobacter nitrofigilis* | DSM 7299 |
| BAK72004.1 | CsdA | 511 | DEAD-box ATP dependent DNA helicase | [G2HRN8](http://www.uniprot.org/uniprot/G2HRN8) | *Arcobacter sp.* | L |
| ABV66327.1 | CsdA | 516 | ATP-dependent RNA helicase, DEAD box family | [A8EQV3](http://www.uniprot.org/uniprot/A8EQV3) | *Arcobacter butzleri* | RM4018 |
| ADR34825.1 | CsdA | 526 | DEAD/DEAH box helicase domain protein | [E4U3C8](http://www.uniprot.org/uniprot/E4U3C8) | *Sulfuricurvum kujiense* | DSM 16994 |
| AFV98356.1 | CsdA | 531 | dead/deah box helicase domain-containing protein | [K7SPP0](http://www.uniprot.org/uniprot/K7SPP0) | *uncultured Sulfuricurvum sp.* | RIFRC-1 |
| ADN08567.1 | CsdA | 518 | DEAD/DEAH box helicase domain protein | [E0UPA3](http://www.uniprot.org/uniprot/E0UPA3) | *Sulfurimonas autotrophica* | DSM 16294 |
| ABB45041.1 | CsdA | 540 | DEAD/DEAH box helicase-like protein | [Q30PP0](http://www.uniprot.org/uniprot/Q30PP0) | *Sulfurimonas denitrificans* | DSM 1251 |
| ADV46511.1 | CsdA2 | 462 | DEAD/DEAH box helicase domain protein | [E6WYS5](http://www.uniprot.org/uniprot/E6WYS5) | *Nitratifractor salsuginis* | DSM 16511 |
| BAF72386.1 | CsdA2 | 460 | ATP-dependent RNA helicase, DEAD-box family | [A6QA77](http://www.uniprot.org/uniprot/A6QA77) | *Sulfurovum sp.* | NBC37-1 |
| AFL69539.1 | CsdA | 590 | DNA/RNA helicase, superfamily II | [I3Y015](http://www.uniprot.org/uniprot/I3Y015) | *Sulfurospirillum barnesii* | SES-3 |
| ACZ13137.1 | CsdA | 583 | DEAD/DEAH box helicase domain protein | [D1B4W7](http://www.uniprot.org/uniprot/D1B4W7) | *Sulfurospirillum deleyianum* | DSM 6946 |
| CAE10536.1 | CsdA | 505 | ATP-DEPENDENT RNA HELICASE, DEAD-BOX FAMILY DEAD | [Q7M8Q7](http://www.uniprot.org/uniprot/Q7M8Q7) | *Wolinella succinogenes* | DSMZ 1740 |
| DEAD | CsdA | 513 | ATP-dependent RNA helicase DeaD | [I2FBI8](http://www.uniprot.org/uniprot/I2FBI8) | *Helicobacter cinaedi* | PAGU611 |
| DEAD | CsdA | 530 | ATP-dependent RNA helicase DeaD | [Q7VFA9](http://www.uniprot.org/uniprot/Q7VFA9) | *Helicobacter hepaticus* | ATCC 51449 |
| DEAD | CsdA | 513 | DEAD box helicase family protein | [D3UJ82](http://www.uniprot.org/uniprot/D3UJ82) | *Helicobacter mustelae* | 12198 |
| CCB79203.1 | CsdA | 470 | cold-shock DEAD-box protein A | [F8KR30](http://www.uniprot.org/uniprot/F8KR30) | *Helicobacter bizzozeronii* | CIII-1 |
| DEAD | CsdA | 469 | ATP-dependent RNA helicase,DEAD box helicase family protein | [E7AD78](http://www.uniprot.org/uniprot/E7AD78) | *Helicobacter felis* | ATCC 49179 |
| AFI04592.1 | CsdA | 513 | ATP-dependent RNA helicase | [I0ENS3](http://www.uniprot.org/uniprot/I0ENS3) | *Helicobacter cetorum* | MIT 00-7128 |
| DEAD | CsdA | 494 | Superfamily II DNA and RNA helicasesATP-dependent RNA helicase | [Q17W71](http://www.uniprot.org/uniprot/Q17W71) | *Helicobacter acinonychis* | Sheeba |
| AAD07315.1 | CsdA | 492 | ATP-dependent RNA helicase, DEAD-box family (deaD) | [O25029](http://www.uniprot.org/uniprot/O25029) | *Helicobacter pylori* | 26695 |
| SRMB | SRMB | 444 | enzyme; RNA synthesis, modification, DNA transcriptionATP-dependent RNA helicase | [P21507](http://www.uniprot.org/uniprot/P21507) | *Escherichia coli* | K-12 |
| RHLB | RHLB | 421 | putative enzyme; Not classifiedATP-dependent RNA helicase | [P0A8J8](http://www.uniprot.org/uniprot/P0A8J8) | *Escherichia coli* | K-12 |
| ADR33941.1 | RhlE1 | 417 | DEAD/DEAH box helicase domain protein | [E4TXN2](http://www.uniprot.org/uniprot/E4TXN2) | *Sulfuricurvum kujiense* | DSM 16994 |
| AFV97365.1 | RhlE1 | 417 | hypothetical protein | [K7S9F7](http://www.uniprot.org/uniprot/K7S9F7) | *uncultured Sulfuricurvum sp.* | RIFRC-1 |
| ADN09231.1 | RhlE1 | 415 | DEAD/DEAH box helicase domain protein | [E0USZ0](http://www.uniprot.org/uniprot/E0USZ0) | *Sulfurimonas autotrophica* | DSM 16294 |
| ABB45219.1 | RhlE1 | 411 | DEAD/DEAH box helicase-like protein | [Q30P62](http://www.uniprot.org/uniprot/Q30P62) | *Sulfurimonas denitrificans* | DSM 1251 |
| AFL67821.1 | RhlE1 | 412 | DNA/RNA helicase, superfamily II | [I3XV47](http://www.uniprot.org/uniprot/I3XV47) | *Sulfurospirillum barnesii* | SES-3 |
| ACZ11502.1 | RhlE1 | 407 | DEAD/DEAH box helicase domain protein | [D1AZN5](http://www.uniprot.org/uniprot/D1AZN5) | *Sulfurospirillum deleyianum* | DSM 6946 |
| ADG93625.1 | RhlE1 | 412 | DEAD/DEAH box helicase domain protein | [D5V013](http://www.uniprot.org/uniprot/D5V013) | *Arcobacter nitrofigilis* | DSM 7299 |
| BAK73084.1 | RhlE1 | 408 | RNA helicase | [G2HVD1](http://www.uniprot.org/uniprot/G2HVD1) | *Arcobacter sp.* | L |
| ABK82754.1 | RhlE? | 624 | putative ATP-dependent RNA helicase RhlE | [A0RP33](http://www.uniprot.org/uniprot/A0RP33) | *Campylobacter fetus* | 82-40 |
| AFL68874.1 | RhlE2 | 430 | DNA/RNA helicase, superfamily II | [I3XY50](http://www.uniprot.org/uniprot/I3XY50) | *Sulfurospirillum barnesii* | SES-3 |
| ACZ12554.1 | RhlE2 | 436 | DEAD/DEAH box helicase domain protein | [D1B384](http://www.uniprot.org/uniprot/D1B384) | *Sulfurospirillum deleyianum* | DSM 6946 |
| BAF71672.1 | RhlE2 | 447 | ATP-dependent RNA helicase | [A6Q863](http://www.uniprot.org/uniprot/A6Q863) | *Sulfurovum sp.* | NBC37-1 |
| ADR33623.1 | RhlE2 | 458 | DEAD/DEAH box helicase domain protein | [E4U2N8](http://www.uniprot.org/uniprot/E4U2N8) | *Sulfuricurvum kujiense* | DSM 16994 |
| AFV97286.1 | RhlE2 | 458 | hypothetical protein | [K7SLL9](http://www.uniprot.org/uniprot/K7SLL9) | *uncultured Sulfuricurvum sp.* | RIFRC-1 |
| ADN09592.1 | RhlE2 | 423 | DEAD/DEAH box helicase domain protein | [E0UV10](http://www.uniprot.org/uniprot/E0UV10) | *Sulfurimonas autotrophica* | DSM 16294 |
| ABB43889.1 | RhlE2 | 432 | DEAD/DEAH box helicase-like protein | [Q30SZ2](http://www.uniprot.org/uniprot/Q30SZ2) | *Sulfurimonas denitrificans* | DSM 1251 |
| ADG93916.1 | RhlE2 | 434 | DEAD/DEAH box helicase domain protein | [D5V0V4](http://www.uniprot.org/uniprot/D5V0V4) | *Arcobacter nitrofigilis* | DSM 7299 |
| BAK73602.1 | RhlE2 | 299 | putative ATP-dependent RNA helicase | [G2HWU9](http://www.uniprot.org/uniprot/G2HWU9) | *Arcobacter sp.* | L |
| ABV67376.1 | RhlE2 | 351 | putative ATP-dependent RNA helicase RhlE | [A8ETV2](http://www.uniprot.org/uniprot/A8ETV2) | *Arcobacter butzleri* | RM4018 |
| RHLE | RHLE | 454 | putative enzyme; Not classifiedATP-dependent RNA helicase | [P25888](http://www.uniprot.org/uniprot/P25888) | *Escherichia coli* | K-12 |
| RHLE | RhlE3 | 457 | ATP-dependent RNA helicase | [A6Q9U1](http://www.uniprot.org/uniprot/A6Q9U1) | *Sulfurovum sp.* | NBC37-1 |
| ADV46755.1 | RhlE3 | 421 | DEAD/DEAH box helicase domain protein | [E6X032](http://www.uniprot.org/uniprot/E6X032) | *Nitratifractor salsuginis* | DSM 16511 |
| ADN09050.1 | RhlE3 | 417 | DEAD/DEAH box helicase domain protein | [E0US27](http://www.uniprot.org/uniprot/E0US27) | *Sulfurimonas autotrophica* | DSM 16294 |
| ABB43306.1 | RhlE3 | 398 | DEAD/DEAH box helicase-like protein | [Q30UM5](http://www.uniprot.org/uniprot/Q30UM5) | *Sulfurimonas denitrificans* | DSM 1251 |
| AFL69226.1 | RhlE3 | 417 | DNA/RNA helicase, superfamily II | [I3XZ52](http://www.uniprot.org/uniprot/I3XZ52) | *Sulfurospirillum barnesii* | SES-3 |
| ACZ12866.1 | RhlE3 | 417 | DEAD/DEAH box helicase domain protein | [D1B446](http://www.uniprot.org/uniprot/D1B446) | *Sulfurospirillum deleyianum* | DSM 6946 |
| ADR34281.1 | RhlE3 | 431 | DEAD/DEAH box helicase domain protein | [E4U095](http://www.uniprot.org/uniprot/E4U095) | *Sulfuricurvum kujiense* | DSM 16994 |
| AFV97121.1 | RhlE3 | 429 | hypothetical protein | [K7SL85](http://www.uniprot.org/uniprot/K7SL85) | *uncultured Sulfuricurvum sp.* | RIFRC-1 |
| ADG94090.1 | RhlE3 | 480 | DEAD/DEAH box helicase domain protein | [D5V1C8](http://www.uniprot.org/uniprot/D5V1C8) | *Arcobacter nitrofigilis* | DSM 7299 |
| BAK73839.1 | RhlE3 | 448 | ATP-dependent RNA helicase | [G2HXI6](http://www.uniprot.org/uniprot/G2HXI6) | *Arcobacter sp.* | L |
| RHLE | RhlE3 | 435 | ATP-dependent RNA helicase RhlE | [A8EUW0](http://www.uniprot.org/uniprot/A8EUW0) | *Arcobacter butzleri* | RM4018 |
