# Supplementary material for: The Sole DEAD-Box RNA Helicase of the Gastric Pathogen Helicobacter pylori Is Essential for Colonization
Source: mBio. 2018 Mar 27;9(2):e02071-17. doi: 10.1128/mBio.02071-17 (PMC5874925; doi:10.1128/mBio.02071-17)
Supplement: TABLE S6 [file mbo001183784st6.docx]

**Supplementary material:**

Table S6 : RNase J proteins

| **Name** | **Length** | **Function** | **SP link** | **Species** | **Strain** |
| --- | --- | --- | --- | --- | --- |
| BAF69179.1 | 608 | conserved hypothetical protein | [A6Q120](http://www.uniprot.org/uniprot/A6Q120) | *Nitratiruptor sp.* | SB155-2 |
| ACM93750.1 | 610 | metallo-beta-lactamase family protein | [B9L716](http://www.uniprot.org/uniprot/B9L716) | *Nautilia profundicola* | AmH |
| ADG91817.1 | 645 | RNA-metabolising metallo-beta-lactamase | [D5V484](http://www.uniprot.org/uniprot/D5V484) | *Arcobacter nitrofigilis* | DSM 7299 |
| BAK72019.1 | 644 | conserved hypothetical protein | [G2HRQ3](http://www.uniprot.org/uniprot/G2HRQ3) | *Arcobacter sp.* | L |
| ABV66337.1 | 641 | conserved hypothetical protein, predicted hydrolase of the metallo-beta-lactamase superfamily | [A8EQW3](http://www.uniprot.org/uniprot/A8EQW3) | *Arcobacter butzleri* | RM4018 |
| ADR32781.1 | 695 | RNA-metabolising metallo-beta-lactamase | [E4TWV0](http://www.uniprot.org/uniprot/E4TWV0) | *Sulfuricurvum kujiense* | DSM 16994 |
| AFV96413.1 | 708 | hypothetical protein | [K7S1K1](http://www.uniprot.org/uniprot/K7S1K1) | *uncultured Sulfuricurvum sp.* | RIFRC-1 |
| ADN10189.1 | 649 | beta-lactamase domain protein | [E0US96](http://www.uniprot.org/uniprot/E0US96) | *Sulfurimonas autotrophica* | DSM 16294 |
| ABB45363.1 | 648 | conserved hypothetical protein | [Q30NR8](http://www.uniprot.org/uniprot/Q30NR8) | *Sulfurimonas denitrificans* | DSM 1251 |
| ADV45333.1 | 693 | RNA-metabolising metallo-beta-lactamase | [E6WY85](http://www.uniprot.org/uniprot/E6WY85) | *Nitratifractor salsuginis* | DSM 16511 |
| BAF73301.1 | 702 | conserved hypothetical protein | [A6QCU2](http://www.uniprot.org/uniprot/A6QCU2) | *Sulfurovum sp.* | NBC37-1 |
| AFL67410.1 | 664 | hypothetical protein | [I3XTY6](http://www.uniprot.org/uniprot/I3XTY6) | *Sulfurospirillum barnesii* | SES-3 |
| ACZ11101.1 | 665 | beta-lactamase domain protein | [D1B0V7](http://www.uniprot.org/uniprot/D1B0V7) | *Sulfurospirillum deleyianum* | DSM 6946 |
| EAT99070.1 | 718 | metallo-beta-lactamase family protein | [A7ZGB5](http://www.uniprot.org/uniprot/A7ZGB5) | *Campylobacter concisus* | 13826 |
| EAT99784.1 | 574 | conserved hypothetical protein | [A7GVW7](http://www.uniprot.org/uniprot/A7GVW7) | *Campylobacter curvus* | 525.92 |
| ABS51684.1 | 678 | metallo-beta-lactamase family protein | [A7I418](http://www.uniprot.org/uniprot/A7I418) | *Campylobacter hominis* | ATCC BAA-381 |
| ABK82291.1 | 646 | metallo-beta-lactamase family protein | [A0RRT5](http://www.uniprot.org/uniprot/A0RRT5) | *Campylobacter fetus* | 82-40 |
| ACM63386.1 | 653 | metallo-beta-lactamase family protein | [B9KEA1](http://www.uniprot.org/uniprot/B9KEA1) | *Campylobacter lari* | RM2100; ATCC BAA-1060D |
| CAL35804.1 | 665 | putative metallo-beta-lactamase family protein | [Q0P7S1](http://www.uniprot.org/uniprot/Q0P7S1) | *Campylobacter jejuni* | NCTC 11168 |
| CAE09235.1 | 682 | conserved hypothetical protein | [Q7MSV9](http://www.uniprot.org/uniprot/Q7MSV9) | *Wolinella succinogenes* | DSMZ 1740 |
| AAP77770.1 | 716 | conserved hypothetical protein | [Q7VGZ4](http://www.uniprot.org/uniprot/Q7VGZ4) | *Helicobacter hepaticus* | ATCC 51449 |
| BAM13209.1 | 713 | putative metallo-beta-lactamase family protein | [I2FEC1](http://www.uniprot.org/uniprot/I2FEC1) | *Helicobacter cinaedi* | PAGU611 |
| CBG39301.1 | 674 | Putative metallo-beta-lactamase | [D3UFN0](http://www.uniprot.org/uniprot/D3UFN0) | *Helicobacter mustelae* | 12198 |
| CCB80259.1 | 627 | Zn-dependent hydrolase, RNA-metabolising | [F8KTT9](http://www.uniprot.org/uniprot/F8KTT9) | *Helicobacter bizzozeronii* | CIII-1 |
| CBY83225.1 | 584 | Putative metallo-beta-lactamase | [E7A8U4](http://www.uniprot.org/uniprot/E7A8U4) | *Helicobacter felis* | ATCC 49179 |
| AFI03652.1 | 702 | ATP/GTP binding protein | [I0EL33](http://www.uniprot.org/uniprot/I0EL33) | *Helicobacter cetorum* | MIT 00-7128 |
| CAJ98968.1 | 689 | Predicted hydrolase of the metallo-beta-lactamase superfamilyconserved hypothetical protein | [Q17ZF8](http://www.uniprot.org/uniprot/Q17ZF8) | *Helicobacter acinonychis* | Sheeba |
| AAD08469.1 | 690 | conserved hypothetical ATP-binding protein | [P56185](http://www.uniprot.org/uniprot/P56185) | *Helicobacter pylori* | 26695 |
